# Recent findings and future directions for interpolar mitotic kinesin inhibitors in cancer therapy

**DOI:** 10.4155/fmc.16.5

**Published:** 2016-03-15

**Authors:** Stephanie M. Myers, Ian Collins

**Affiliations:** Cancer Research UK Cancer Therapeutics Unit, The Institute of Cancer Research, London SM2 5NG, U.K

**Keywords:** Kinesin, Eg5, HSET, Mitosis, Allosteric, Inhibition, Resistance

## Abstract

The kinesin class of microtubule-associated motor proteins present attractive anti-cancer targets owing to their roles in key functions in dividing cells. Two interpolar mitotic kinesins Eg5 and HSET have opposing motor functions in mitotic spindle assembly with respect to microtubule movement, but both offer opportunities to develop cancer selective therapeutic agents. Here, we summarize the progress to date in developing inhibitors of Eg5 and HSET, with an emphasis on structural biology insights into the binding modes of allosteric inhibitors, compound selectivity and mechanisms of action of different chemical scaffolds. We discuss translation of preclinical studies to clinical experience with Eg5 inhibitors, recent findings on potential resistance mechanisms, and explore the implications for future anticancer drug development against these targets.

## 1 Interpolar mitotic kinesins: Function and structure

Chemotherapeutic agents which target microtubules have been used to treat various cancer types. Microtubules are formed from the polymerisation of tubulin monomers, and microtubule-targeting drugs may hyper-stabilise GDP-bound polymerised tubulin, for example taxanes and epothilones, or act by destabilising and depolymerising microtubules, for example vinca alkaloids and colchicine. Both classes of molecule hinder the dynamic processes of microtubule formation and remodelling. This is particularly important in cell division, where tubulin polymerisation and depolymerisation is essential for the formation of a normal mitotic spindle [1]. Although clinically successful, the significant neuropathic side-effects associated with the use of microtubule-targeting agents that act as mitotic spindle poisons is dose limiting. Furthermore, resistance to these drugs is common [2,3]. Alternative strategies to specifically target the mitotic spindle in cancer cells have been explored. The kinesin class of microtubule-associated motor proteins represent attractive anti-cancer targets owing to their roles in key functions in dividing cells.

### The Role of Kinesins in Mitosis

The kinesin superfamily consists of over 650 distinct proteins which are categorized into 14 subfamilies according to their structural similarity and function. Over 70 of these proteins belong to the kinesin-5 family, and are found in eukaryotic organisms [4]. The mitotic kinesins (in collaboration with the dyneins, another important family of microtubule-associated motor proteins) are integral force generators in the process of cell division, ensuring that chromosomes are separated with the highest integrity [5]. Kinesins use ATP hydrolysis to generate movement along microtubules by means of conformational changes in the protein [6]. Their relatively straightforward yet varying individual roles in spindle dynamics result collectively in complex behaviour [7].

**Figure 1:**
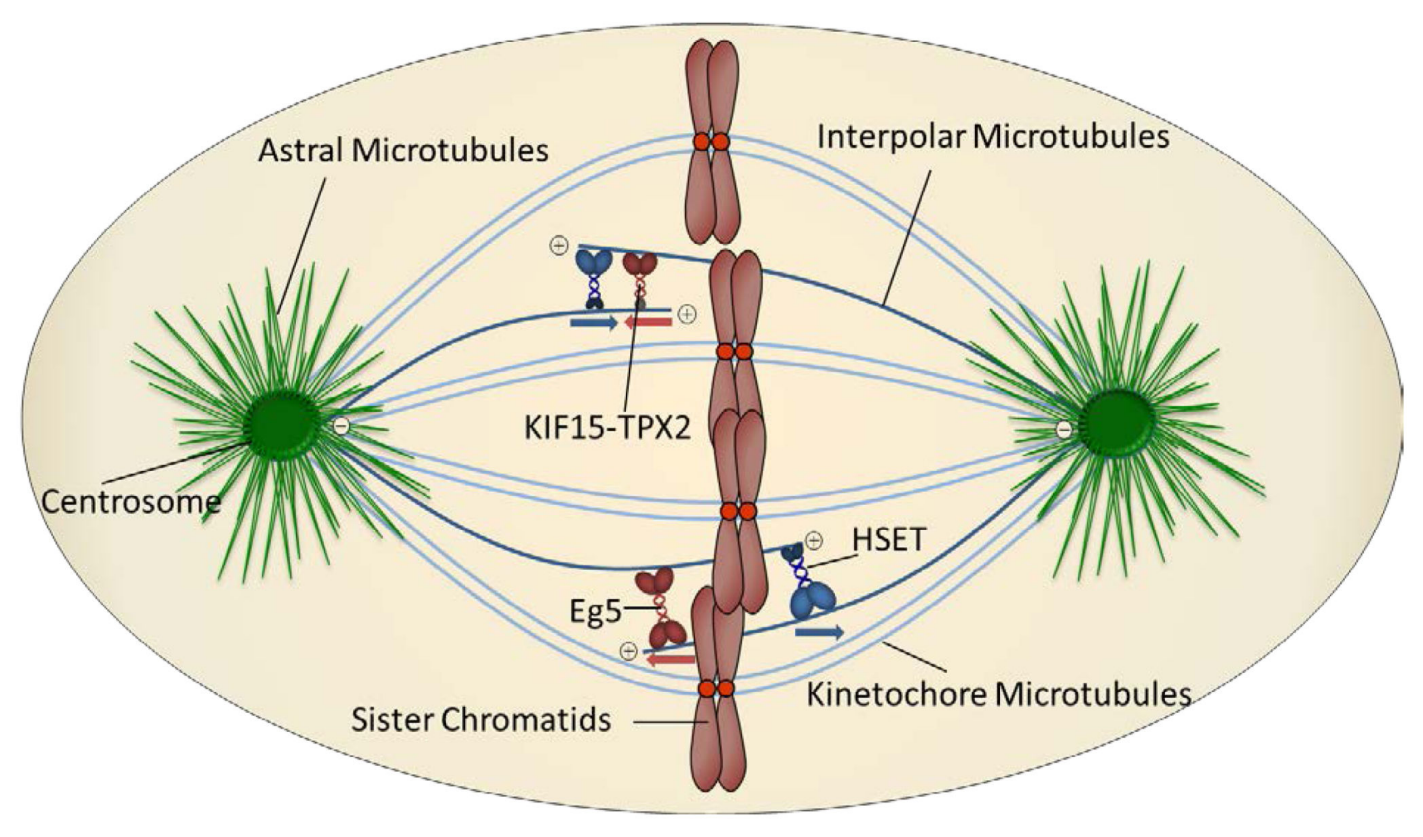
Simplified representation of the mitotic spindle architecture and relevant mitotic kinesins located on intrapolar microtubules. Adapted from reference [8]

There are currently 16 kinesins implicated in coordinating aspects of mitosis and cytokinesis [8]. In this review, we focus on two mitotic kinesins which are located primarily on the interpolar microtubules. These structures extend from one spindle pole across the cell equator and interdigitate with opposing interpolar microtubules, exerting additional pull on the chromosomes thus stabilising the mitotic spindle [9]. The most extensively studied of the mitotic kinesins is Eg5 (also known as KIF11, kinesin-5 or KSP) and is involved in formation of the bipolar spindle, which requires sustained outward forces for its maintenance [10]. Eg5 is thought to exist as a homotetramer which crosslinks two opposing interpolar microtubules, pushing them apart as a result of its plus-end directed motility [8]. The closely related plusend directed kinesin KIF15 has been shown to act cooperatively with Eg5 to promote bipolar spindle assembly [11,12]. In contrast, HSET (also known as KIFC1) is a minus-end directed motor, acting antagonistically towards Eg5 but whose role is also in bipolar spindle assembly [13–15]. HSET has also been implicated in microtubule organisation and promoting stability of the central spindle, a requirement for cytokinesis to be performed correctly (Figure 1) [16].

### 1.2 The Structure of Kinesins

**Figure 2:**
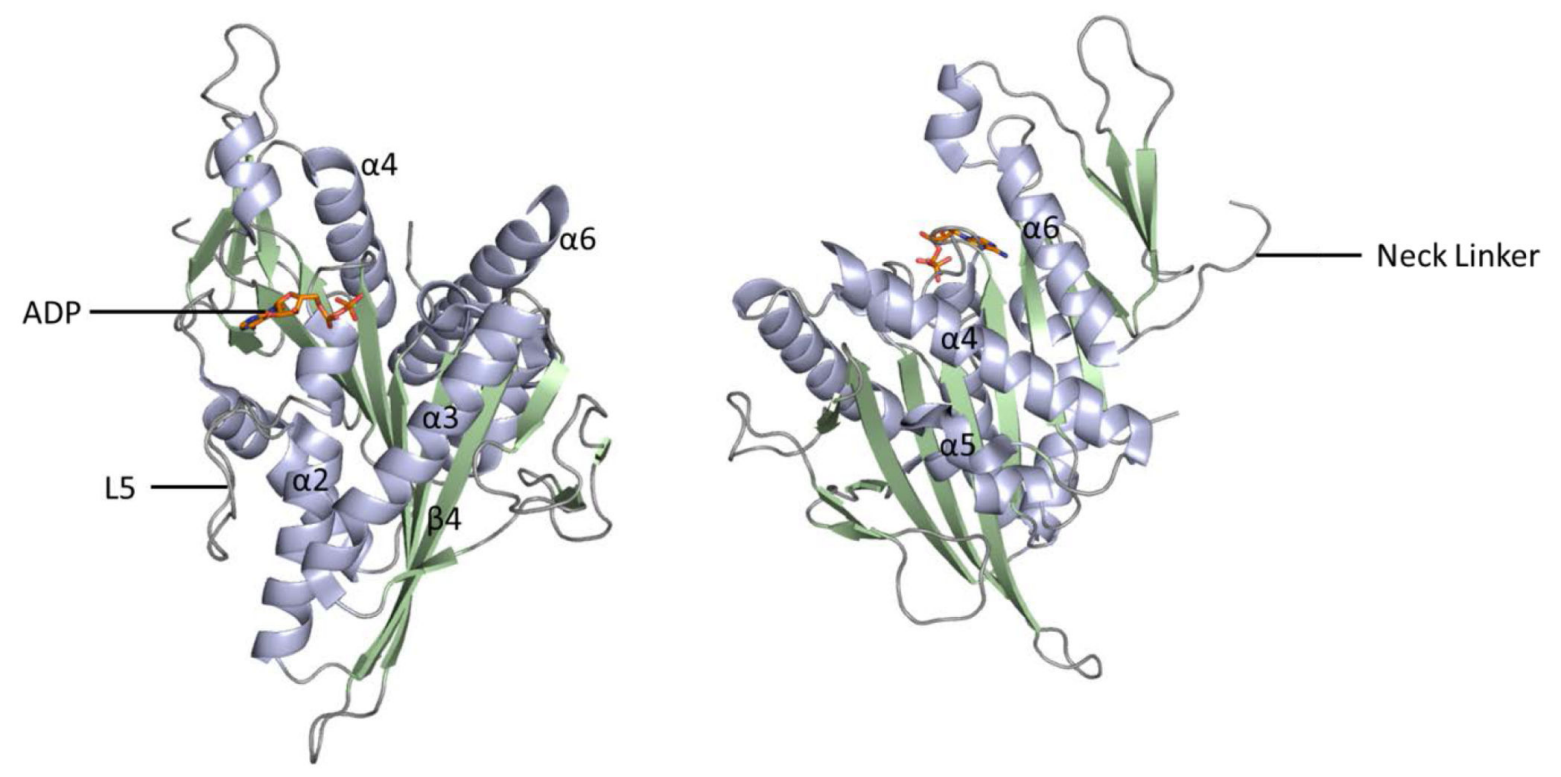
Crystal structure of Eg5-ADP-Mg (PDB 1II6); two alternative views. α helices shown in light blue, β sheets in pale green and loop regions in grey. Bound ADP shown in stick form (orange).

Understanding the structure and function of a protein target to underpin the development of clinical candidates is pivotal in modern drug discovery. Throughout, we will make reference to the key structural features of the Eg5 and HSET kinesins, as illustrated in the Eg5-ADP-Mg crystal structure (Figure 2), with particular emphasis on experimentally determined or proposed inhibitor-bound states.

All cytoskeletal motors, including kinesins, consist of a catalytic motor domain (head) which contains two binding domains, one for ATP and one for microtubules. Kinesins can be classified into N-type kinesins where the motor domain is at the N-terminus (e.g. Eg5), M-type where the motor domain is flanked by other domains, or C-type where the motor domain is close to the C-terminus (e.g. HSET) [17]. Kinesins typically have two globular motor domains, attached to a coiled-coil stalk (neck) region by neck-linkers. At the opposite end of the stalk, is a tail region which is responsible for interacting with specific cargos and adaptor proteins [18].

**Figure 3:**
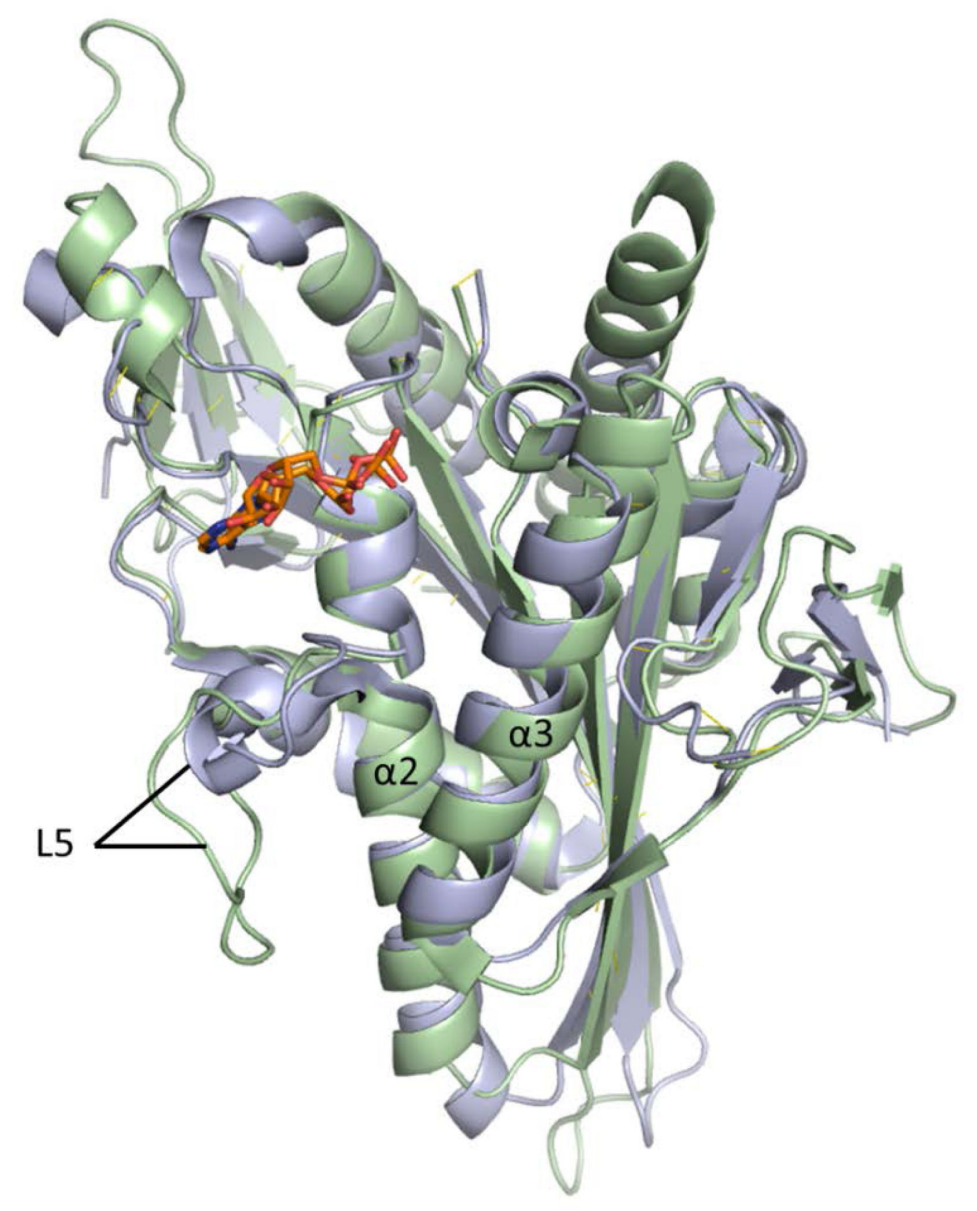
Overlay of crystal structures of Eg5 (PDB 1II6; light green) and HSET (PDB 2REP; blue). Bound ADP shown in stick form (orange). Notable differences in the length of Loop 5 element are evident.

The motor domains of the kinesin superfamily, consisting of ca. 350 amino acids, are highly conserved and thus Eg5 and HSET bear strong structural resemblance in these regions despite their opposing mitotic functions. Eg5 consists of eight antiparallel β strands at its core, flanked by three major α helices on either side. The catalytic site is situated above the central β sheet, surrounded by the phosphate binding loop (P-loop; between α2 and β3) which tightly binds the β-phosphate group of ATP/ADP, and switch motifs I (between α3 and β6) and II (between α4 and β7) which change their conformation depending on the presence or absence of a γ-phosphate group[17,19]. The α2 helix next to the P-loop is unusual; the helical repeat is intersected by the surface-exposed loop 5 that has been proposed to modulate Eg5 function by interacting with the catalytic site. The loop 5 motif is common to both HSET and Eg5, but the difference in amino acid chain length is striking (Figure 3). While the loop 5 element of Eg5 consists of 17 amino acids [20], it has been reported that kinesin-14 family members can have as few as three [21].

At the C-terminus of the motor domain, the neck linker connects to the stalk domain and adopts an immobilised ‘docked’ conformation when the motor domain is bound to microtubules. However, in the absence of microtubules it reverts to a more mobile ‘undocked’ conformation. The neck linker enables Eg5 movement in the direction of the plus end of the microtubule by extending towards it, driving motility in this direction [22]. Notably, in the HSET-ADP-Mg crystal structure, the neck linker is unresolved; suggesting a high degree of flexibility in its nucleotide bound state. However, it has been suggested that the ability of the neck-linker to dock is reliant on residues conserved only in the Eg5 subfamily [20]. This reversible process is enabled by a cluster of secondary structures which consists of α4, L12 and α5 and is connected to switch II by L11, which in the ATP-bound state adopts a conformation which is amenable to neck linker docking [17].

## 2 Eg5 and HSET as Therapeutic Targets

### 2.1 Eg5 Target Validation

Owing to their roles in cell division, Eg5 and HSET both represent potential cancer-selective therapeutic targets. Eg5 is reportedly selectively overexpressed in several tumour types, including those of the breast, colon, lung, ovary and uterus [23]. An association of high Eg5 expression and poor clinical outcome has been established in several cancer types including non-muscle invasive bladder urothelial carcinoma, [24] renal cell carcinoma [25] and pancreatic adenocarcinoma [26]. Transgenic mice overexpressing Eg5 suffer chromosome missegregation, genomic instability, and have a higher incidence of tumour formation compared to control animals [27]. Depletion of Eg5 using endonuclease-prepared siRNA (esiRNA) in HeLa cells distinctively showed perturbation of bipolar spindle formation causing cells to exhibit monopolar spindles, termed ‘monoasters’ [14]. Similarly, depletion of Eg5 using alternative methods such as antisense oligomers (ASO) or siRNA in other cell lines decreased cell proliferation and increased apoptotic cell death [28]. Targeting Eg5 with siRNA has been shown to kill tetraploid cells more efficiently than diploid precursor cells, suggesting that clinical Eg5 inhibition may provide a window of selectivity for cancer cells over normal cells, thus minimising side-effects [29]. Depletion of Eg5 using ASO treatment in CaP and LNCaP prostate cancer cell lines reduced both Eg5 mRNA and Eg5 protein levels, resulting in dose-dependent growth inhibition, G2/M arrest and apoptosis. Interestingly, low dose Eg5-ASO seemed to antagonise the cytotoxic effects of paclitaxel. *In vivo*, ASO monotherapy decreased both CaP and LNCaP tumour growth, but combination treatment with paclitaxel did not elicit an additive response [30]. *In vivo* knockdown of Eg5 was also effective in UG87 glioblastoma and MDA-MB-231 breast cancer [28], ovarian cancer and melanoma [31] and EPP85 pancreatic tumour xenograft models [26].

Eg5 was found to be highly expressed in blast-crisis chronic myelogenous leukaemia (BC-CML) patient samples, and cell lines which were Philadelphia chromosome-positive. Inhibition of the Bcr-Abl tyrosine kinase by imatinib was shown to downregulate Eg5 expression in imatinib-sensitive, but not in imatinib-resistant or kinase-negative, cell lines [32]. However, knockdown of Eg5 using ASO technology induced G2/M arrest and cell death in both imatinib-sensitive and resistant cell lines, suggesting that an Eg5 inhibitor could be used clinically in patients who have developed resistance to Bcr-Abl kinase inhibitors [32]. Eg5 expression in patients with non-small cell lung cancer (NSCLC) was correlated with cyclin B1 expression and appeared to be predictive of improved clinical response to antimitotic agents in combination with platinum therapy. In this study, 37% of Eg5-positive patients showed a clinical response to treatment, compared with only 10% of Eg5-negative patients [33].

### 2.2 HSET Target Validation

While esiRNA-mediated depletion of HSET also resulted in perturbation of bipolar spindle formation, the effect was significantly different to that observed following Eg5 depletion as, in contrast to the distinctive monoaster formation, HeLa cells treated with HSET esiRNA exhibited multipolar spindles [14]. In HeLa cells, HSET siRNA resulted in formation of truncated spindles, but did not affect pole formation [15]. However, HSET depletion in breast cancer cell lines indicated that centrosome amplified cells were particularly sensitive, since they exhibited a higher frequency of multipolar spindles compared with non-centrosome amplified controls [34]. HSET is believed to play a key role in the survival of centrosome amplified cancer cells, enabling formation of a pseudo-bipolar spindle through clustering supernumerary centrosomes, which allows the cells to evade apoptotic mechanisms at the mitotic checkpoint [35]. Owing to this role in centrosome clustering, clinical inhibition of HSET may provide a therapy for centrosome-amplified tumours.

HSET siRNA induced multipolar mitoses in breast cancer and melanoma cell lines selectively over non-transformed cells. However, the proportion of cancer cells with multipolarity did not significantly correlate with the percentage of supernumerary centrosomes. Additionally, HSET depletion did not enhance the frequency of supernumerary centrosomes. This data suggested that HSET may be involved in bipolar spindle formation in cancer cells irrespective of centrosome number [36]. A recent study which characterised the expression of HSET in numerous human breast cancer cell lines showed that HSET was highly expressed in all eight tested, but was undetectable in human normal mammary epithelial cells (HMEC). Furthermore, siRNA-mediated knockdown of HSET in two of the cancer cell lines confirmed a reduction in cell viability following treatment [37].

Clinically, HSET overexpression has been correlated with poor prognosis in breast cancer [37] and ovarian adenocarcinoma patients [38]. Elevated HSET gene expression has been detected in numerous other cancer types, including glioblastoma, lung, breast, colon and cervical tumour samples, in comparison to corresponding normal tissues [39]. Additionally, in NSCLC HSET expression was found to be highly predictive of the presence of brain metastasis in both early and advanced disease [40].

## 3 Eg5 Chemical Probes: Structural Findings

Chemical inhibitors are invaluable tools for the deconvolution of biological processes and validation of novel molecular targets. The two chemical probes monastrol and (*S*)-trityl-*L*-cysteine (STLC) which specifically target Eg5 have been extensively studied, and have provided important information regarding the biological function of this interesting kinesin, as well as structural information and insights into the mechano-chemistry of kinesins.

### 3.1 Representative Structures of Eg5 Inhibitors

To date, numerous compounds showing inhibitory activity against Eg5 have been identified, and these have been recently reviewed [41]. In this article, we will focus on the Eg5 inhibitors with reported crystallographic data on their binding modes (Table 1).

### 3.2 Monastrol and Analogues

**Figure 4:**
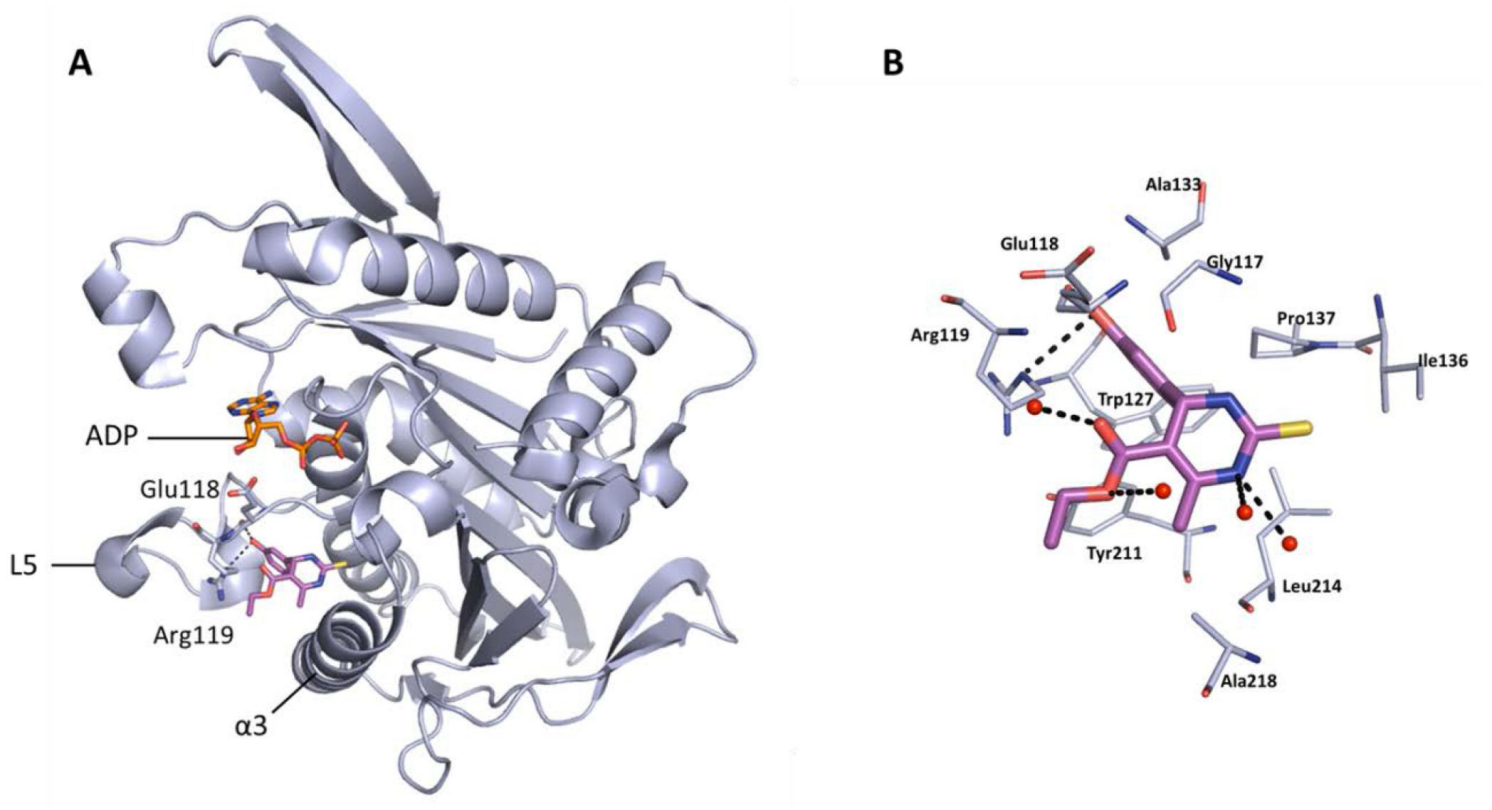
Binding site of monastrol (sticks shown in magenta) (PDB 1X88), located in the L5/α2/α3 allosteric pocket of ADP-bound Eg5. A) Hydrogen bonds between the phenolic OH with Glu118 (2.7 Å) and Arg119 (3.3Å) are shown. Bound ADP shown in stick form (orange). B) Detailed binding site view of monastrol bound in the L5/α2/α3 allosteric pocket. The dihydropyrimidinethione core of monastrol makes several hydrophobic interactions with a surface consisting of the side-chains of residues Gly117, Ile136, Pro137, Tyr211, Leu214, and Ala218. A second hydrophobic cleft made up of Arg119, Trp127, Ala133 and Tyr211 houses the phenol ring. Key hydrogen bonds are shown between the phenolic OH and the carbonyl of the backbone of Glu118, and between the oxygen of the phenol and the guanidine side-chain of Arg119. Additional hydrogen bonding interactions made with water molecules (red) *via* the ester motif and NH of the dihydropyrimidinethione core are also shown.

Monastrol was the first chemical probe to be identified as an inhibitor of Eg5, and was discovered by a phenotypic screening approach described in 1999 [43]. Characterised as an ATP non-competitive, reversible inhibitor, monastrol was found to induce a ‘mono-astral’ phenotype in cells, leading to mitotic arrest. Kinetic studies indicated that binding of an inhibitor such as monastrol to the Eg5-ADP complex prevented the force generation and kinesin motility by two modes: Firstly, the release of ADP from the protein is inhibited, preventing completion of the catalytic cycle. Secondly, the conformational state upon binding has a lower affinity for microtubules, the scaffolding required for bipolar spindle formation [52]. Successful co-crystallisation of the inhibitor-bound Eg5 complex in 2004 confirmed the allosteric site of inhibition to be a pocket residing between Loop 5 and the α2 and α3 helices of the protein, situated 12Å away from the nucleotide binding site (Figure 4a) [53].

The crystal structure of (*S*)-monastrol (**1**), the more potent of the two enantiomers, bound to Eg5 highlighted a number of key interactions, which are shown in detail in Figure 4b. The majority of Eg5 inhibitors described to date occupy similar areas within the L5/α2/α3 pocket, including all of those which have reached clinical trials.

**Figure 5:**
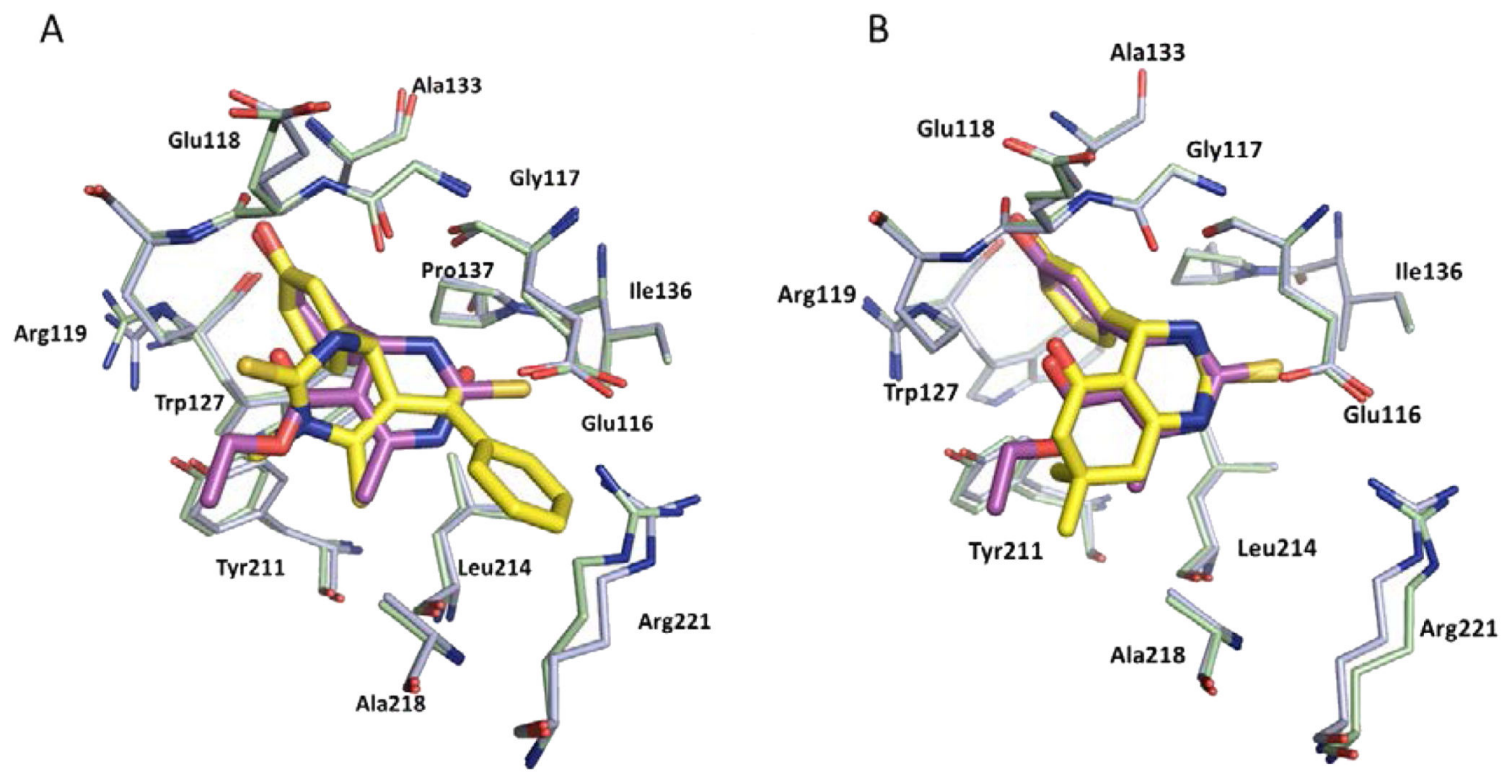
A) Overlay of Monastrol (**1**) (magenta, PDB 1X88, light blue ADP-Eg5) and (*R*)-Mon-97, **2(*R***) (yellow, PDB 2IEH, pale green ADP-Eg5) showing the ‘flipped’ binding orientation. Hydrogen bonds between the phenolic OH of **2(*R***) with the backbone of Glu118 (2.6Å) and a weaker interaction with the side chain of Arg119 (3.4Å) analogous to those formed by the phenolic OH of monastrol exist but are not shown. An additional hydrogen bond between the NH of the dihydropyrimidinethione core and Gly117 (2.6Å) is also formed; B) Overlay of Monastrol (**1**) (magenta, PDB 1X88, light blue ADP-Eg5) and (*S*)-dimethylenastron (**4**) (yellow, PDB 2X7D, pale green ADP-Eg5). The two ligands bind in the same orientation and hydrogen bonds to the protein are identical.

A structurally-related analogue of monastrol bound to the same allosteric site, but the opposite enantiomer, (*R*)-Mon-97 **2(*R*)**, showed higher affinity and thus preferentially co-crystallised in the allosteric site of ADP-bound Eg5. (*R*)-Mon-97 differs from monastrol in that the ethyl ester is replaced with a bulky acetophenone group, and one of the nitrogen atoms of the dihydropyrimidinethione motif is methylated. These subtle structural changes resulted in a ‘flipped’ binding orientation which placed the lipophilic phenyl ring of the acetophenone group in the hydrophobic cleft previously occupied by the dihydropyrimidine core of monastrol, and the thioxo group pointing towards the solvent-exposed surface, making a network of hydrogen-bond interactions with two water molecules [44]. Interestingly, the (*S*)-enantiomer **2(*S***) was also reasonably potent against Eg5 which warns against the assumption that a single enantiomer will be solely responsible for on-target potency across a given chemical series binding in this site, possibly because the protein is amenable to significant conformational changes. Remarkably, despite the differences in binding mode, the overall protein structures of **1-** and **2(*R***)-bound Eg5 are very similar, resulting in identical modes of action (Figure 5a) [45].

Efforts to improve the biochemical and cellular potency of dihydropyrimidinethione-based inhibitors resulted in the discovery of enastron (**3**), dimethylenastron (**4**) and fluorastrol (**5**). Crystallographic studies revealed a similar phenomenon with these structurally related dihydropyrimidinethiones, whereby enastron and dimethylenastron bind primarily in the (*S*)-configuration (Figure 5b), while the (*R*)-enantiomer of fluorastrol binds preferentially. The 3-phenol group is common to all three inhibitors, and was shown to be in the same position in all three crystal structures. The additional potency gains in enastron and dimethylenastron were attributed to the reduced flexibility of the molecules, and a better fit into the solvent exposed sub-pocket of Eg5. Despite pointing towards solvent, the dimethyl groups of dimethylenastron maintain hydrophobic contacts with the main chain of Ala218, and significantly, one methyl group makes a CH-π interaction with Tyr211. The five-fold increased cellular potency of (*R*)-fluorastrol relative to (*R*)-Mon-97 in terms of growth inhibition in a HCT116 cell line (see Table 1) has been attributed to the ability of one of the fluorine atoms to form multipolar interactions within the binding site. Additionally, the electron rich fluorine atoms are in close proximity to a positively charged Arg221 residue, and the resulting electron deficient aromatic ring which makes a π-stacking interaction with the salt bridge formed by Glu116 and Arg221 is more favourably positioned near to the negatively charged carboxylate of Glu116, which could be partly responsible for the potency gains observed in this assay [44]. However, since no biochemical assay data is available for (*R*)-fluorastrol, it is not possible to determine whether all of the observed cellular potency gains are a direct result of improved ligand-protein interactions. Other aspects such as differences in cell permeability as a result of increased inhibitor lipophilicity, and equilibria between different conformational states of Eg5 could also be contributing to the differences in cell potency.

The flexibility of Eg5 to accommodate structurally-related compounds in opposite binding modes highlights the caveats in using *in silico* drug-design predictive tools against highly mobile proteins such as kinesins.

While tetrahydro-*β*-carboline inhibitors (Table 1, compounds **6-9**) were discovered independently in a high-throughput screening campaign and are a structurally distinct compound class, these inhibitors were found to occupy the same binding region as the dihydropyrimidinethione based analogues [45]. Hit compound **6** was identified as a modestly potent inhibitor of Eg5 and incorporation of a methyl group to give **7** improved potency tenfold, presumably by increasing hydrophobic contacts in the lipophilic Leu214 region. While the compounds were screened as racemates, the (*R*)-enantiomer of **7** bound preferentially to Eg5 (PDB 3K3B, not shown), and the crystal structure revealed that the phenolic motif makes a hydrogen bonding interaction with Glu118 (2.6Å), thus mimicking the OH group of monastrol. A second hydrogen bond is evident between the NH of the tetrahydro-*β*-carboline core and Glu116 (3.1Å) and a third is identified between the amide carbonyl lone pair and the main-chain amide NH of Arg119, although with suboptimal geometry. The potency of **7** was increased further by incorporating a basic side-chain (**8**), which was hypothesised to interact with Glu215 or Tyr211, although not crystallographically confirmed. Interestingly, the phenol motif which presents a metabolic liability for further development of this series could be successfully replaced with a fluorophenyl group (**9**) without significantly compromising potency [46]. Since a structure of **9** bound to Eg5 has not been reported, the reasons why this compound retains a high level of potency remain unclear. An alternative binding orientation as seen with the dihydropyrimidine-based analogues, or a pseudo hydrogen-bonding interaction between the fluorine atom and the key Glu118 of Eg5 could be possible explanations.

### 3.3 (S)-Trityl-L-Cysteine (STLC) and Analogues

STLC (**10**) was identified as an ATP-non-competitive and reversible inhibitor of Eg5 in both basal and microtubule-stimulated biochemical assays. Additionally, STLC caused mitotic arrest in HeLa cells. The binding site of STLC is the same as that of monastrol, occupying the L5/α2/α3 allosteric pocket. However, STLC had much tighter binding than monastrol, owing to an eight-fold faster association rate and four-fold slower release rate [47].

**Figure 6:**
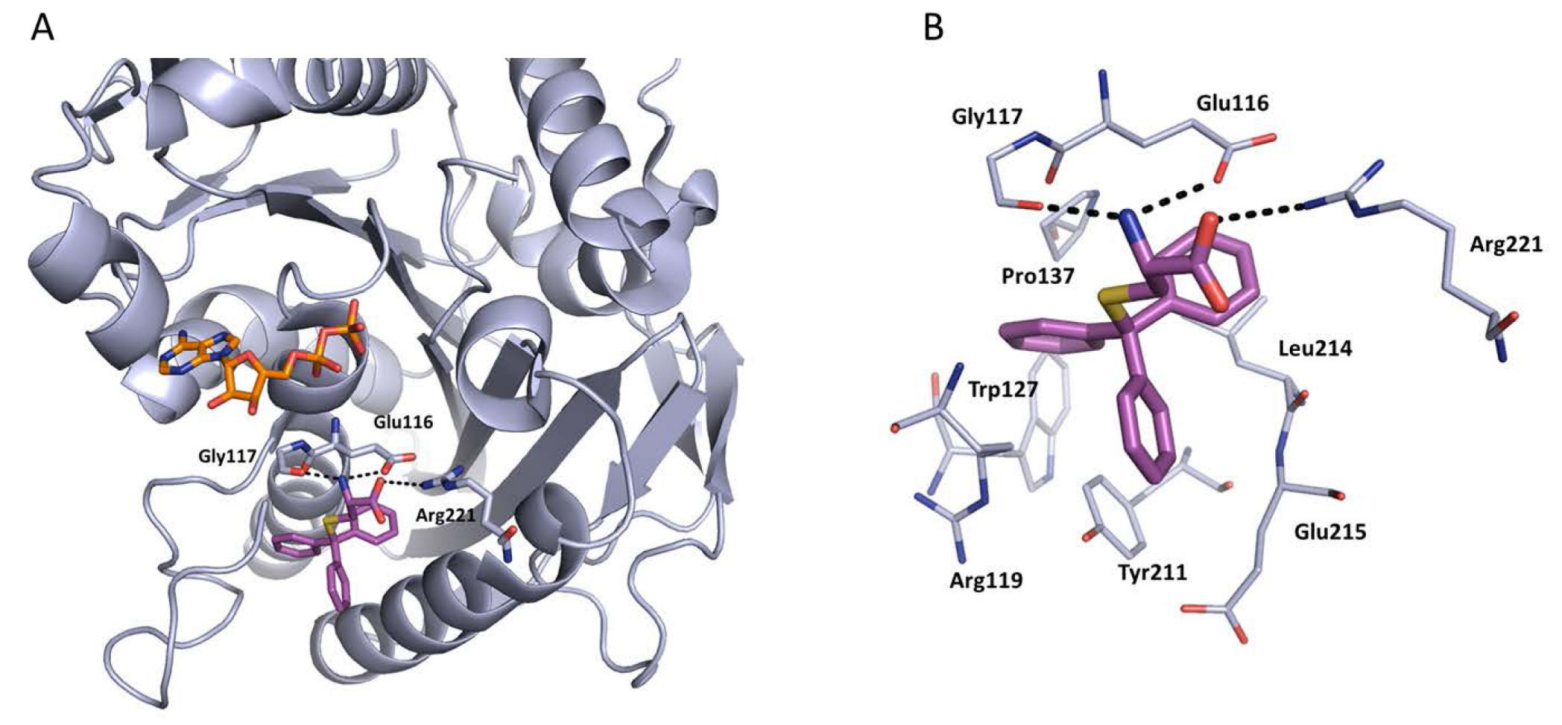
The binding interactions of STLC (magenta, PDB 3KEN) with ADP-Eg5 (light blue). A) Polar interactions are made between the amine of STLC with the side-chain of Glu116 (3.2 Å) and the main-chain of Gly117 (2.7 Å), and the acid of STLC forms a hydrogen bond with Arg221 (3.6 Å) (dotted lines). Bound ADP is shown in stick form (orange). B) Detailed binding site view of STLC bound in the L5/α2/α3 allosteric pocket. The lipophilic triad of phenyl rings in STLC makes several hydrophobic interactions with the alkyl side-chains of residues Glu215, Glu116 and Arg119, anchoring the compound into the binding site. Several aromatic stacking interactions are evident from the crystal structure including an edge-to-face interaction with Trp127 and a CH-π interaction between Pro137 and the first phenyl ring. The second phenyl ring makes an offset stacked π-π interaction with Tyr211, and the third aromatic ring makes another CH-π type interaction with the aliphatic side-chain of Leu214. The polar side-chain of STLC is exposed to solvent, making hydrogen bonds between the residues shown, and water molecules (not shown).

Crystallographic studies showed that STLC makes a number of hydrophobic, π-stacking and hydrogen bonding interactions with Eg5 (Figure 6). Most strikingly, not only were the interactions between STLC and Eg5 revealed from the co-crystallisation studies, but the sequence of Eg5 conformational changes upon inhibitor binding was elucidated, as an intermediate state of the kinesin-inhibitor complex was serendipitously captured [54].

Opportunities for the optimisation of STLC-based inhibitors have been exploited, to improve upon biochemical, cellular and *in vivo* activity. Incorporation of small substituents at one or more positions on the rotationally interchangeable phenyl rings increased potency by maximising hydrophobic interactions in the core of the protein e.g. (*S*)-methoxytrityl-*L*-cysteine (**11**), while bulkier hydrophobic substituents were not productive as the trityl group rotates to place the larger substituent in the solvent exposed sub-pocket [48,55,56]. Removal of the metabolically labile sulfur atom and isosteric replacement with a carbon linkage gave a series of triphenylbutanamines [49,57] exemplified by compound **12** which showed significant tumour growth inhibition in a range of cancer cell lines (HCT116, LNCaP, K562, PC3, BxPC-3 and NCI-HI299) and gave regressions of a subcutaneous tumour xenograft of LXFS538 lung cancer cells following an IP dosing schedule [49].

More recently, conformationally restrained tricyclic analogues of STLC have been reported, which sought to increase the hydrophobic van der Waals interactions observed between STLC and the protein while reducing entropy. Indeed, ring closure of (*S*)-methoxytrityl-*L*-cysteine with an ethylene linker to give a seven-membered fused carbocycle (**13**) displayed a twelve-fold improvement in biochemical potency and an eight-fold improvement in cytotoxicity in the HCT116 cell line. *In vivo* activity in an HCT116 colon cancer xenograft model following a 25 mg/kg IV dose was also reported [50].

### 3.4 The Pathway of Eg5 Structural Changes elucidated using chemical tools

The binding of an Eg5 inhibitor results in a wide variety of drug-induced structural changes, and elucidation of these effects has been of considerable interest. While crystallographic snapshots of monastrol and its analogues bound to Eg5 provided insight into the mode of action of inhibition, how these changes were initiated and how subsequent conformational changes were connected remained unclear.

**Figure 7:**
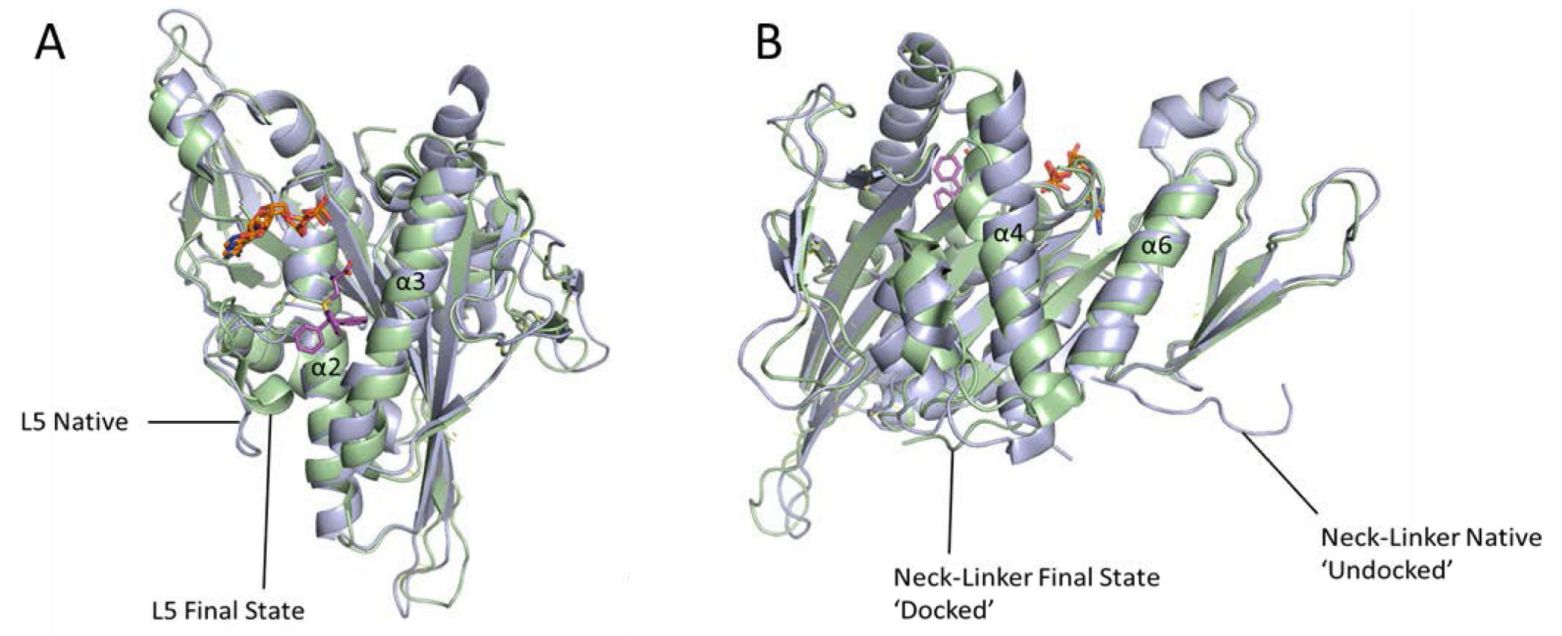
The sequence of conformational changes induced upon inhibitor binding to Eg5. Overlays of native ADP-Eg5 (light blue, PDB 1II6) with the crystal structure of STLC-ADP-Eg5 in its final inhibitor bound state (pale green, PDB 2WOG). Bound ADPs (orange) and STLC (magenta) are shown in stick form. A) Loop L5 in native Eg5 swings round to close the inhibitor binding pocket, and helix α3 has shifted. B) The switch II cluster (helix α4, loop L12 and helix α5) is shifted to a ‘permissive’ conformation which has allowed the neck linker to dock onto the microtubule-binding face of Eg5.

Studies using Forster resonance energy transfer (FRET) indicated a biphasic conformational change of the Eg5 motor domain. The first phase occurs rapidly and involves movements within the inhibitor binding site, including the residue Trp127 which was monitored for quenching by monastrol binding. The second much slower phase seemed to result in the docking of the neck-linker onto the motor domain [58,59]. The crystallographically-determined intermediate state of STLC-bound Eg5 corroborated the conclusions drawn from FRET studies on the intermediate transition states involved (Figures 7a and 7b), confirming that binding of STLC causes drug-induced transition through three distinct stages; 1. The loop L5 in native Eg5 swings round to close the inhibitor binding pocket, 2. The switch II cluster (helix α4, Loop L12 and helix α5) rearranges to adopt a ‘permissive’ conformation, opening up space for the final stage, 3. The neck-linker docks onto the motor domain to give the final inhibitor-bound complex [54]. As Eg5 has a unique, extended loop 5, it is currently unknown whether kinesins with shorter L5 motifs such as HSET exhibit similar conformational changes upon inhibition.

## 4 HSET inhibitors as chemical tools

### 4.1 CW069

While previously considered a less tractable target than Eg5, the recent emergence of two selective chemical probes for this motor protein has provided exciting opportunities to address the potential role for HSET inhibitors in cancer therapy. In the absence of any available structurally characterised chemical tools, CW069 (**17**) was identified using a ‘chemogenomics-based’ compound selection approach, using the principle that similar proteins bind will similar ligands [60,62,63]. An *in silico* model for HSET binding was developed, based on the existing inhibitors of Eg5 mined from the CHEMBL database. Owing to the high degree of sequence similarity (80%) in the motor domains of Eg5 and HSET it was rationalised that inhibitors of HSET binding L5/α2/α3 would be identified using this approach. Biochemical profiling of a triaged selection of 50 compounds led to the discovery of two inhibitors, one of which, containing a γ-lactone benzoic acid moiety, was selective for HSET over Eg5. Hit expansion led to the discovery of CW069 which inhibits HSET selectively with modest biochemical potency. Of 64 analogues tested, only CW069 displayed any activity against HSET, indicating a narrow structure-activity relationship window for this chemotype.

A ligand-based binding mode prediction (using PDB files 2REP and 1I16 with CW069 plus one other analogue) gave calculated protein-ligand interaction enthalpic energies for HSET and Eg5 which were consistent with the selectivity observed against Eg5. The model predicted a key hydrogen bond interaction between Arg521 and the carboxylate of CW069, which appeared to be responsible for the selectivity observed between HSET and Eg5, where the analogous residue is Ala218. Additional proposed interactions included; hydrogen-bond interactions between backbone CO and NH of Gly423, Leu517 and the carboxylate and amine groups of CW069. Interestingly, a structurally similar analogue of CW069 was not HSET selective, which was rationalised using molecular dynamics data for the protein. Despite the shorter chain length of loop 5 in HSET relative to Eg5, HSET L5 was found to be extremely dynamic, particularly around a glycine rich motif which is not present in Eg5. This resulted in opening of the binding pocket, allowing CW069 to be accommodated, whereas Eg5 consistently showed partial closure of the analogous binding space. Despite its weak biochemical activity, CW069 was shown to increase multipolar spindle formation in breast cancer cell lines containing supernumerary centrosomes (MDA-MB-231 and BT549) without altering bipolar spindle formation in a non-centrosome amplified cell line [60].

### 4.2 AZ82

High-throughput screening of 800,000 compounds followed by subsequent iterative medicinal chemistry was used to identify the potent HSET inhibitor AZ82 (**18**). Impressively, over 1500 compounds were profiled within one week using an integrated HTS, synthesis and screening campaign to enable rapid optimisation of the chemical series. An HSET selective inhibitor with no activity against a panel of nine other kinesins including Eg5, AZ82 bound to the HSET/microtubule binary complex and inhibited microtubule-stimulated HSET ATPase activity. Co-sedimentation analysis revealed AZ82 to be ATP/ADP competitive, stabilising an HSET state with a higher affinity for microtubules, an opposite mode of action from the Eg5 inhibitor monastrol which inhibits ADP release from the protein and results in an Eg5 conformational state with a lower binding affinity for microtubules. Equilibrium dialysis/mass spectrometry (ED/MS) and fluorescent nucleotide exchange experiments also suggested that AZ82 bound to the HSET/microtubule complex, not HSET or microtubules alone [61,64].

Unfortunately, no crystal structure of HSET with an inhibitor bound has yet been solved, and ligand-protein complex crystallography was noted to prove difficult in the case of AZ82 despite its high affinity for the target. This is possibly due to the requirement for HSET to be in the microtubule-bound state to bind AZ82. In order to gain insights into the binding mode of this inhibitor, a homology model based on available kinesin-inhibitor structures was constructed. Owing to the structural similarity of AZ82 to GSK923295, a compound which binds in the analogous L5/α2/α3 allosteric binding pocket of the structurally related kinesin CENP-E, the model incorporated aspects of the inhibited CENP-E protein [65] as well as the crystal structures of Eg5 bound to the clinical candidate EMD-534085 [66] and ADP-bound HSET. The homology model suggested that the thiophene ring was buried deeply in the hydrophobic pocket lined with aromatic rings of Tyr461, Phe542 and the aliphatic chain of Glu421. The trifluoromethyl moiety of the biaryl group was positioned in a pocket formed by the L5 loop, and the polar pyrrolidine tail was directed towards the nucleotide binding site [61,64]. The ability of AZ82 to reverse the monopolar spindle phenotype observed following treatment with an Eg5 inhibitor and cause centrosome declustering in centrosome amplified cancer cell lines provided further evidence that inhibition of HSET presents an alternative opportunity for pharmacological modulation of the motor protein function.

## 5 Eg5 inhibitor clinical candidates

The Eg5 inhibitors which have been evaluated in clinical trials to date are ATP noncompetitive and target the L5/α2/α3 allosteric pocket (Table 3). Despite nine inhibitors reaching the clinic, all are based around only three broad chemical scaffolds, with several overlapping structural features (although the structure of 4SC-205 is undisclosed). Since these clinical candidates target the same binding site as that of monastrol and STLC, a common mode of inhibition is shared. The discovery and development of Eg5 inhibitors has been reviewed in detail elsewhere [41,67,68] and therefore this section will summarize the key features of the binding modes and the clinical outcomes to date.

### 5.1 Binding features of Eg5 clinical candidates

**Figure 8:**
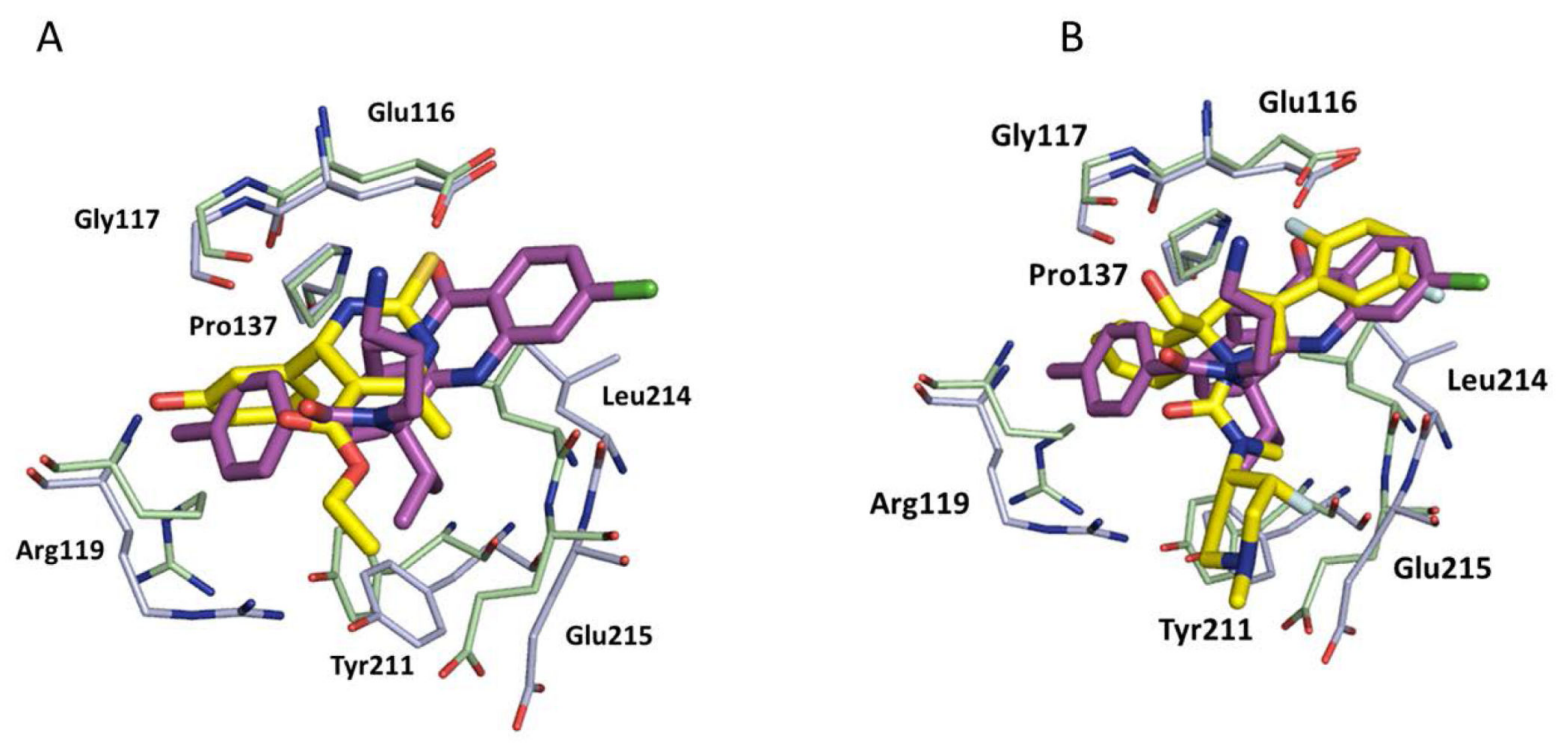
A) Overlay of the binding of ispinesib (**19**) (magenta ligand, light blue ADP-Eg5, PDB 4AP0) and monastrol, **1** (yellow ligand, pale green ADP-Eg5, PDB 1X88). Ispinesib induces a larger binding pocket than monastrol, owing to a 1.3Å shift of the Leu214 backbone to accommodate the benzyl group, which makes stacking interactions with Pro137 and Tyr211. Tyr211 and Glu215 are also shifted by 1-2Å relative to monastrol to accommodate both the benzyl group and the aromatic ring of the benzamide moiety. The methyl benzamide group mimics the phenol ring of monastrol, almost overlapping, but the side-chain of Arg119 must shift over 3Å to accommodate this group. The flexible alkylamino chain in ispinesib forms a network of hydrogen bonds by directly interacting with the carboxylate of Glu116, which in turn makes a hydrogen-bond with Gly117 *via* a water molecule (not shown). B) Overlay of the binding of ispinesib (**19**) (magenta ligand, light blue ADP-Eg5, PDB 4AP0) and the clinical candidate MK-0731 (**24**) (yellow ligand, pale green ADP-Eg5, PDB 3CJO). The difluorophenyl motif of MK-0731 overlays well with the chloroquinazolinone core of ispinesib but MK-0731 makes an interaction with Gly117 rather than Glu116. While the basic side chain of Arg119 is pushed aside upon ispinesib binding, it is able to fold atop MK-0731, making a favourable π-stacking interaction in doing so [75,76].

Despite the fact that ispinesib (**19**) was the first to reach the clinic, and the quinazolinone-based scaffold of this inhibitor dominates current clinical candidates, little is reported regarding the first discovery of this chemotype and its subsequent development. A family of quinazolinone-containing analogues were identified following a high-throughput screening campaign, which displayed promising initial properties including IC_50_ values below 1 μM, as well as sub-10 μM *in vitro* potency in cells and greater than 100-fold selectivity over a panel of twelve other kinesins. Key areas of optimisation for both potency and cell permeability included the length and basicity of the ethylamine side-chain, the requirement for 4-substitution on the benzamide group and the required substitution and stereochemistry at the α-amino group. The resulting candidate compound ispinesib had Ki < 1 nM, GI_50_ < 1 nM in the SKOV3 human ovarian carcinoma cell line, good aqueous solubility and oral bioavailability [70]. Many of the structural features of ispinesib are present in the second-generation inhibitor SB-743921 (**20**), with the core quinazolinone heterocycle being replaced by a chromenone. This subtle change improved activity against Eg5 giving a remarkably potent inhibitor (Ki = 0.1 nM) with dose-dependent activity in a number of tumour xenograft models [71]. Other investigations led to the discovery of AZD-4877 (**21,** IC_50_ = 2 nM), also in clinical development, where the quinazoline is replaced by an isothiazolo[5,4-d]pyrimidin-4-one [72]. While the quinazolinone Arq621 (**22**) has more structurally diverse substituents, many of the key features of ispinesib are retained. There is no crystallographic data reported for Arq621 at present, but it is assumed that the binding mode will be similar to that of ispinesib [73]. The majority of ispinesib’s potency is likely due to the extensive hydrophobic interactions, inducing a larger pocket than that of monastrol (Figure 8a) [74,75]. Remarkably, only one direct hydrogen-bonding interaction is consistently made with the protein, as it is observed in three of the four chains within the ternary complex. In the fourth chain of the ternary complex, the aminopropyl side-chain is disordered, highlighting its flexibility.

High-throughput screening has proven to be a successful technique for the identification of Eg5 inhibitor scaffolds, with subsequent optimisation leading to the discovery of the other clinical candidates; hexahydro-2*H*-pyrano[3,2-c]quinolone (HHPQ) compound EMD-534085 (**23**) [66] and the 2,5-dihydropyrrole MK-0731 (**24**) [77,78]. The clinical candidates ARRY-520, **25** [79] and LY2523355, **26** [80] bear structural resemblance to MK-0731, but replace the 2,5-dihydropyrrole core with a 1,3,4-thiadiazoline motif. Despite the structural diversity between EMD-534085, MK-0731 and ispinesib, crystallography revealed several overlapping features (Figure 8b). The thiophene-containing chemical probes **14, 15** and **16** (Table 1) also bind similarly to MK-0731 and were identified *via* a HTS approach [51].

**Figure 9:**
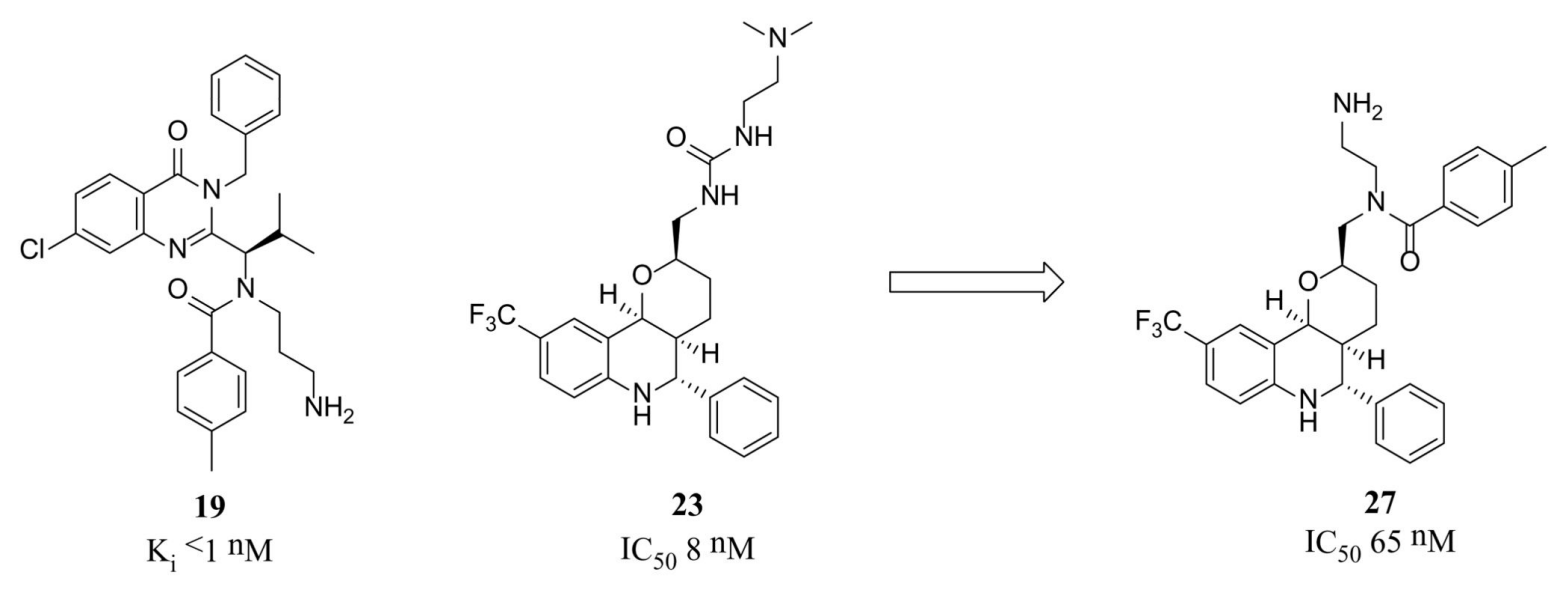
Scaffold-hopping approach towards an EMD-534085 (**23**)-ispinesib (**19**) hybrid compound **27** [66].

The C-9 trifluoromethyl substituent of EMD-534085 also occupies a position analogous to the chloroquinazolinone moiety of ispinesib, but the basic side-chain of EMD-534085 serves to increase aqueous solubility of the lipophilic core, pointing towards solvent. This improves the metabolic stability and pharmacokinetic properties of the lipophilic core, but is not reported to make any binding interactions with the protein. Additionally, a putative HHPQ-ispinesib hybrid compound (**27**) was eight-fold less active than EMD-534085, supporting the differences in binding modes (Figure 9) [66].

### 5.2 Clinical outcomes with Eg5 inhibitors

Despite all Eg5 inhibitors reaching clinical trials displaying low nanomolar biochemical potency and good cellular activity against the target, clinical data has been disappointing to date, as many Eg5 inhibitors have failed to show efficacy as a monotherapy. One of the exceptions is ARRY-520 (**25**) (filanesib), which has demonstrated clinical activity in patients with relapsed or refractory multiple myeloma [81]. According to the product pipelines available on the relevant pharmaceutical company websites (accessed October 2015), only two compounds appeared to be reported as in clinical development; ARRY-520 and 4SC-205 (Table 3). The reasons for the poor activity of many Eg5 inhibitors in the clinic are unclear, although several hypotheses have been proposed.

It has been postulated that the clinical effectiveness of classical microtubule-targeted agents in general may be due in part to the disruption of the microtubule-associated functions of non-dividing tumour cells [82], or through inhibition of angiogenesis by host vascular cells [83]. Eg5 is almost exclusively expressed during the mitotic phase of the cell cycle, although some is expressed in G2. As a result, drugs targeting Eg5 may only have a therapeutic effect on cells which are undergoing cell division at the time of treatment [84]. Since preclinical models based on tumour xenograft have faster cell cycling rates, the preclinical results may not be sufficiently realistic as predictors of clinical success [49]. This could also apply to the development of inhibitors of other kinesins whose expression is limited to M-phase. Interestingly, recent clinical data using the oral Eg5 inhibitor 4SC-205 indicated that a continuous dosing scheme of 20 mg per patient, once daily could overcome this ‘proliferation rate paradox’. A clinical response (stable disease) was observed in 67% of patients for more than 100 days undergoing this regimen [85].

A secondly challenge to clinical efficacy is possible functional redundancy amongst mitotic kinesins. Primarily involved in maintenance of the bipolar spindle [8], KIF15 (kinesin-12) has been proposed as a potential compensatory kinesin for Eg5. Evidence to suggest that KIF15 drives bipolar spindle assembly in the absence of Eg5, compensating for its inhibition when over-expressed, has been reported [11]. Interest in this functionally-related kinesin has led to its biochemical characterisation and comparison to Eg5. In contrast to Eg5, KIF15 does not contain a second nucleotide-independent microtubule-binding site in its C-terminal tail, and operates as a dimer to cross-link kinetochore microtubules, as opposed to the homotetrameric structure of Eg5. Owing to the structural differences between the two kinesins, microtubule binding is most likely carried out by the targeting protein for Xklp2 (TPX2), which complexes to KIF15 through its C-terminus, promoting centrosome separation. Interestingly, TPX2 is also known to interact with the mitotic kinase Aurora A, and the sensitivity of non-Hodgkin lymphoma (NHL) cell lines to the Aurora A inhibitor MK-8745 was increased when TPX2 was depleted with siRNA [86,87]. It has therefore been proposed that KIF15 and its protein-protein interaction with TPX2 represent interesting therapeutic targets, perhaps with utility in combination with an Eg5 inhibitor [11,88,89]. Clinical use of drug combinations are a valid chemotherapeutic strategy, which have been shown to result in synergistic effects in some cases, and offer a greater chance of overcoming acquired drug resistance [90]. The effectiveness of the Eg5 candidate SB-743921 was enhanced when dosed in combination with the Aurora-A kinase inhibitor alisertib, and restored activity in a drug-resistant cell line [91].

A third, mechano-chemical, explanation for resistance to Eg5 inhibition is proposed. It has been demonstrated that the flexible loop L5 which is coupled to the nucleotide binding site and the neck-linker element which initiates forward motility, serve to accelerate ADP release during the initial microtubule binding event of the catalytic cycle. However, the observation that a seven amino acid deletion within L5 can still hydrolyse ATP suggests that L5 is not essential to promote subsequent movement along the microtubules. While inhibitors targeting the L5/α2/α3 allosteric site are known to kinetically slow the release of ADP, doubts have arisen regarding how effectively mitosis can be inhibited by these kinetically slowed but non-arrested motors, particularly since multiple motors are engaged to separate centrosomes biologically [92]. This suggests the development of Eg5 inhibitors with an alternative mode of action, e.g. ATP-competitive, may present a potential avenue for investigation to remedy the low clinical efficacy.

## 6 Resistance to kinesin inhibitors

A major challenge in the area of cancer chemotherapy is the development of drug resistance. Known resistance mechanisms to kinesins can include upregulation of alternative pathways (as discussed in section 5.2), expression of efflux pumps as seen with the CENP-E inhibitor GSK923295 [93], or mutations within the target protein. The emergence of point-mutations conferring resistance to Eg5 inhibitors targeting the L5/α2/α3 site has already been reported [94–97], representing another challenge in the clinical application of Eg5 inhibitors.

### 6.1 Resistance to Chemical Tools

Studies have shown that mutations in the induced-fit binding pocket of Eg5 can confer drug resistance to the inhibitors STLC and monastrol, both biochemically [96] and in a cellular context [97]. The introduction of several single point mutations by alanine scanning mutagenesis led to resistance or partial resistance to monastrol and/or STLC, strongly suggesting that many different residues are equally important for drug binding. Three mutant strains (R119A, D130A and L214A; See online appendix Figure 1) conferred significant resistance to both chemical probes in cell-based assays, characterised by the ability of cells to form normal bipolar spindles [97]. Structural explanations have been offered as to why these three particular residues appear to be important for effective inhibitor binding. Arg119 is located at the entrance of the binding pocket, and forms part of the pocket to which elements of both monastrol (phenol ring) and STLC (trityl motif) make hydrophobic interactions. The side-chains of Asp130 (loop 5) and Leu214 (helix α3) point towards both inhibitors, making favourable hydrophobic contacts or CH-π interactions (refer to sections 3.2 and 3.3 for monastrol and STLC binding modes, respectively). Curiously, these three drug-resistant strains can confer resistance to monastrol and STLC in the presence of wild-type Eg5, suggesting that an Eg5 tetramer consisting of a combination of drug-sensitive and drug-resistant motor domains may still be able to generate force and motility [97]. The phenomenon of drug sensitive proteins pairing with insensitive ones to give a functional oligomer is well documented in other contexts, for example the transactivation of Raf kinase isoform heterodimers upon inhibitor binding resulting in paradoxical activation of the MAPK signalling pathway in cells expressing wild-type B-Raf [98,99]. Encouragingly, the development of ‘paradox-breaking’ pan-Raf inhibitors has been reported [100]. This suggests that if it were possible to develop an inhibitor targeting multiple mutants of Eg5, this could overcome this mechanism of kinesin resistance.

Eg5 mutants resistant to clinical candidates have been identified, highlighting a significant risk of the emergence of resistance in patients treated with an Eg5 inhibitor. Long-term exposure of HCT116 colorectal cell lines to the clinical candidate ispinesib generated an ispinesib-resistant cell line which was >3000-fold less sensitive to the drug, but not cross-resistant to other cytotoxic drug including tubulin-targeting agents [101]. DNA sequencing of the Eg5 motor domain from the resistant cell line identified the two key point-mutations conferring resistance as D130V (also one of the point-mutation residues conferring resistance to monastrol and STLC) and A133D. Potency determinations for ispinesib against the two mutant strains of Eg5 revealed that the D130V is the more resistant of the two [102]. The ability of the mutations to confer resistance was rationalised as a result of their effect on the integrity of the binding pocket. D130 and A133 are involved in an extensive network of hydrogen bond interactions, which has the effect of stabilising the loop L5 in an inward conformation essential for inhibitor binding [102]. These same mutations have been found to also confer resistance to the second generation Eg5 inhibitor SB-743921.

Further investigation of the resistance mechanism using calorimetry showed that mutated forms of Eg5 had improved enthalpic interactions with SB-743921, which is in contrast to the common assumption that mutations which result in drug-resistance involve steric or electrostatic repulsion, leading to an enthalpically less favourable complex. The crystal structure of inhibitor-bound Eg5 A133D mutant did not reveal any obvious repulsive interactions compared with that of the wild-type protein [95].

The use of calorimetry in combination with modelling studies proposed that the resistance of Eg5 towards SB-743921 was through reduced flexibility of the protein as a result of local rearrangement of the hydrogen bonding interactions and salt-bridging in the allosteric pocket. In the wild-type protein Arg138, Ala133, and Asp130 form a network of hydrogen bonds and there is a salt bridge which exists between Glu128 and Lys207 [95,103]. In the A133D variant, Arg138 formed a salt bridge with the newly introduced carboxylate of Asp133, breaking the bridge between Glu128 and Lys207 and allowing a new hydrogen bond interaction to form between Lys207 and His141. These changes rigidify the overall complex. Reduced flexibility is typically associated with an entropy penalty which could account for some of the reduced affinity of the ligand towards Eg5. Additionally, key residues which are most efficient in energy exchange with their surroundings act as ‘energy gates’ [104], which communicate information *via* correlated residue fluctuations. In wild-type Eg5, residue A133 was identified as such an energy gate – able to transmit a perturbation through the protein to the nucleotide site when binding the inhibitor. When A133 was mutated, the energy gate was no longer present in the allosteric pocket, preventing transmission of allosteric inhibition. Crucially, energy gates within the nucleotide site are unaffected, enabling normal ATP hydrolysis and thus Eg5 motor function. This unpredictable phenomenon has been termed ‘resistance by allostery’ [95,103]. The key residues affected by point mutations in the L5/α2/α3 binding site are highlighted in online appendix Figure 1. The authors of this study recommended that if a mutation arises which produces allosteric resistance, the focus should not be on identifying a new inhibitor of the same site, but identifying an alternative binding site with an alternative mode of inhibition to overcome the problem [95].

Since the development of inhibitors of HSET lags those of Eg5, and the binding mode of current inhibitors is unknown, it is not yet possible to determine whether HSET is susceptible to such ‘resistance by allostery’. However, given the high degree of structural similarity between the kinesin families, similar phenomena may be likely to occur. Identifying where and how inhibitors bind and which residues are susceptible to inhibitor-induced mutations is likely to be important for the development of HSET inhibitors as well as for future Eg5 inhibitors.

The mutant strains of Eg5 may prove to be useful screening tools in the discovery of future Eg5 inhibitors. The STLC-resistant cell lines D130A and L214A have shown utility in distinguishing inhibitors which bind to alternative pockets from the classical L5/α2/α3 targeted inhibitors without the requirement of existing structural information. These cell lines were shown to be sensitive to a series of ATP-competitive inhibitors which have been hypothesised to bind in a novel allosteric site [105].

## 7 An alternative allosteric site

The ‘resistance by allostery’ effect observed with SB-743921 led to the suggestion that an inhibitor targeting a novel site would resolve this [95]. Additionally, given the doubts regarding the effectiveness of Eg5 L5/α2/α3 allosteric inhibitors [92], the identification of novel inhibitors with an alternative mode of action is attractive. Intriguingly, a second allosteric site within Eg5 has been identified, with chemical tools co-crystallised in the pocket between the α4 and α6 helices (Table 4).

### 7.1 Biphenyl-Based Inhibitors

In 2007 a set of biphenyl-based inhibitors of Eg5 were identified following a high-throughput screening campaign. Iterative medicinal chemistry to improve potency led to the identification of the highly potent inhibitors GSK-1 (**28**) and GSK-2 (**29**). Further characterisation of these analogues revealed that they were first-in-class ATP-competitive inhibitors of Eg5 [106]. GSK-1 and GSK-2 bound to the ispinesib-resistant D130V and A133D mutants of Eg5, indicating that a binding site distinct to that of previous inhibitors was occupied. GSK-1 also showed potent activity in the HCT116 colon cancer cell line (IC_50_ = 36 nM) giving a phenotype indistinguishable from the classical monastrol phenotype despite the alternative mode of action.

A combination of biochemical and biophysical techniques were used to determine the inhibitor binding site. Preparation of a moderately active photo labile analogue containing a reactive phenyltrifluoromethyldiazirine motif (GSK-3, **30**) allowed covalent modification of the Eg5 protein. Labelled Eg5 motor domain was purified from the required microtubules, and subsequently digested with trypsin. Analysis by MALDI-TOF revealed residues 284-297 as the site of ligand incorporation, and further interpretation of the data specifically identified Leu295 as the site of labelling. Leu295 is located in the middle of helix α4, with its side chain pointing towards the α4/6 interface, and molecular dynamics studies revealed that a small pocket between the switch II α4/6 helices can develop, indicating that despite being ATP-competitive, the binding mode is distinct from the nucleotide binding site [106].

**Figure 10:**
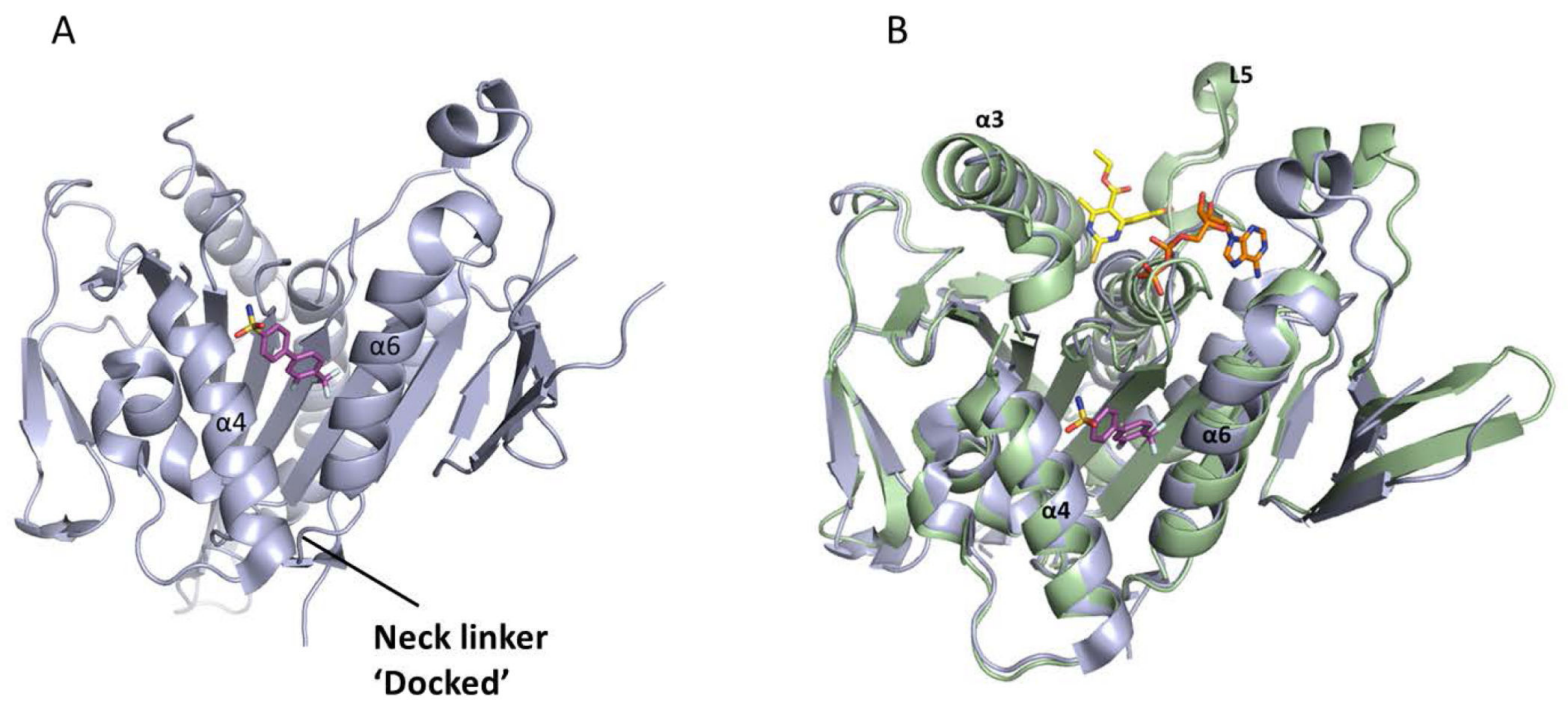
A) Crystal structure of the ATP-competitive Eg5 inhibitor PVZB1194 (**31,** magenta), bound in the α4/α6 allosteric site (PDB 3WPN). The neck-linker is in a docked conformation. B) Overlay of PVZB1194 (**31,** magenta), bound in the α4/α6 allosteric site (light blue, PDB 3WPN) with the ATP non-competitive inhibitor monastrol (**1,** yellow) bound in the L5/α2/α3 allosteric site (pale green, PDB 1X88). Bound ADP shown in stick form (orange).

A range of biphenyl based inhibitors with similar mode of action were investigated independently, resulting in the identification of PVZB1194 (**31**), an ATP-competitive inhibitor of Eg5 [107]. PVZB1194 was eventually successfully co-crystallised with nucleotide-unbound Eg5 (Figure 10a; See Figure 10b for overlay with monastrol-ADP-bound Eg5), owing to the ability of this ligand to bind to Eg5 without the presence of microtubules. The phenyl ring of Tyr104 formed a stacking interaction with the 3-fluoro, 4-trifluoromethylphenyl ring, while Tyr352 stacked with the sulfonamide phenyl group, which made additional van der Waals interactions with Leu292 and Leu293. A network of hydrogen bonds existed between Tyr104, Glu345 and Ser269 which formed the wall of the narrow hydrophobic binding pocket. Finally, the trifluoromethyl group made a series of van der Waals interactions with Tyr104, Ile332, Ala334, Tyr352 and Ala353 towards the bottom of the binding cleft. Interestingly, despite the high degree of potency for PVZB1194 for Eg5, there were no direct hydrogen bonding interactions observed between ligand and protein [111].

The crystal structure showed that the neck-linker was in a docked conformation, which appeared to stabilise the bottom of the PVZB1194-binding pocket. The importance of the neck-linker conformation for PVZB1194 binding was investigated using a deletion mutant, with the neck linker absent from the structure. While the ATPase function was retained, PVZB1194 was found to be forty-fold less potent against this truncated variant, confirming the requirement of a neck-linker-docked conformation for inhibitor binding. Consistent with the biochemical result that the biphenyl-based compounds were ATP-competitive, the nucleotide binding site was void of ATP or ADP. The surface model of the ATP-binding site showed that there was insufficient space to accommodate the nucleotide, as a result of Glu129 and Thr107 occupying the site. Binding of PVZB1194 caused Tyr104 of strand β3, which forms part of the bottom of the inhibitor pocket, to move through 1.8Å which in turn caused Thr107 to shift by 6.8Å, altering the conformation of the ATP-binding site and preventing nucleotide binding.

The novel mode of action of biphenyl-based Eg5 inhibitors has stimulated further research. Improvements in the potency of the ATP-competitive class of inhibitors were achieved by introducing conformational restriction about the biphenyl junction, through development of carboline and carbazole scaffolds, resulting in reduced entropy loss (e.g. **32**) [108]. Unfortunately the planarity of the carbazole compounds resulted in very poor aqueous solubility, rendering the series unsuitable for further development. Ring opening to give a diarylamine-based scaffold gave equipotent ATP-competitive compounds with improved solubility (e.g. **33**) [109].

### 7.2 Benzimidazole-based inhibitors

In 2010, the binding mode of a novel set of substituted benzimidazoles shown to target Eg5 was reported. The ability of these compounds to bind to a novel site was proposed after it was observed that they bound to Eg5 in the presence of ispinesib. Direct binding to D130V, A133D and the D130V-A133D double Eg5 mutants was also observed, further strengthening the hypothesis that a second independent binding site was targeted. The benzimidazole compounds were initially shown to be ATP non-competitive, which is in contrast to the biphenyl second-site binders [110]. Confirmation of the binding site for the aminobenzimidazole-based compound BI8 (**34**) was accomplished with the aid of a crystal structure, published in 2013 [112].

**Figure 11:**
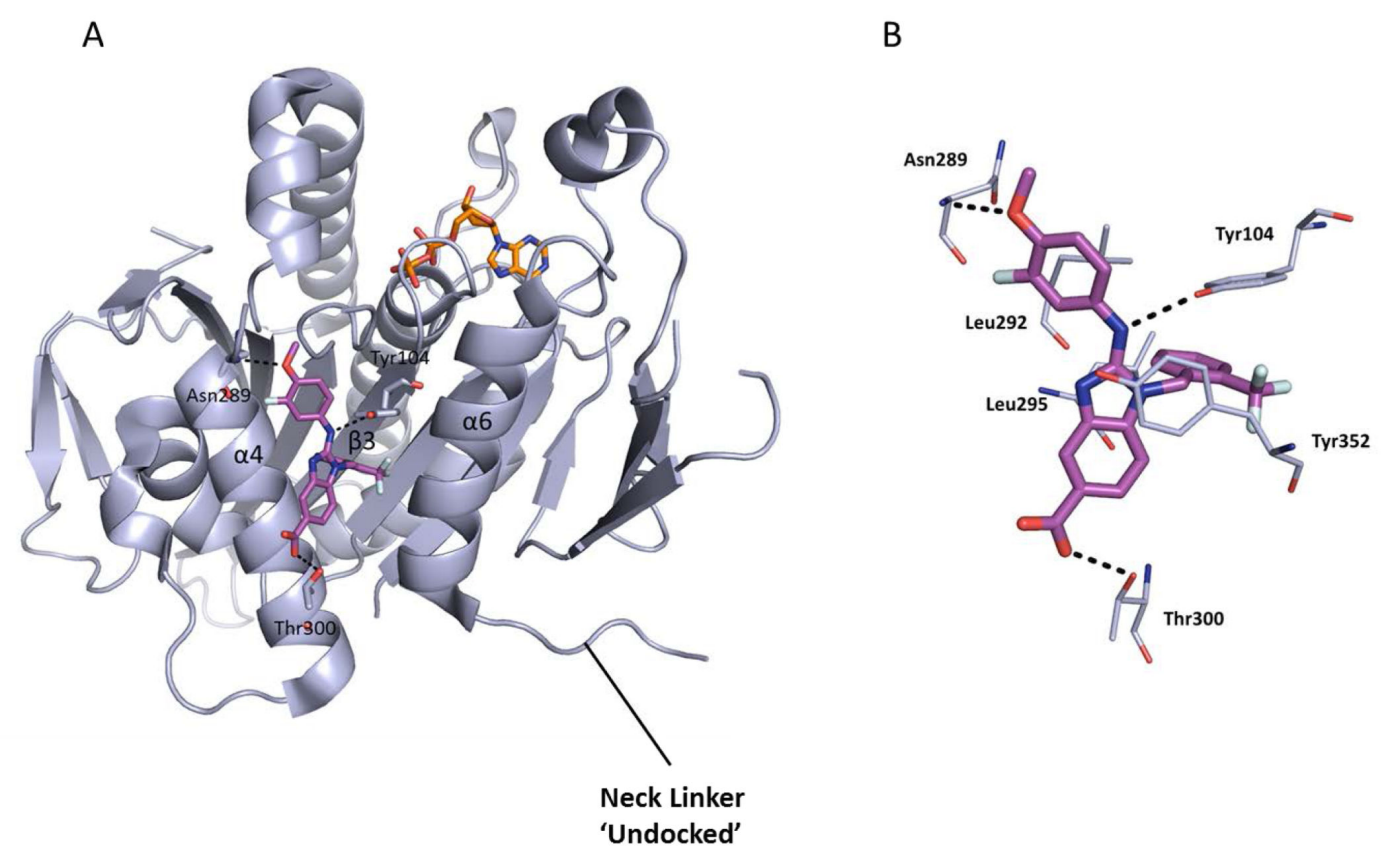
A) Crystal structure of the ATP non-competitive inhibitor BI8 (**34,** magenta) bound in the α4/α6 allosteric site (PDB 3ZCW). Water molecules are not shown. The necklinker is in an undocked conformation and ADP (orange sticks) can be seen in the nucleotide binding site. Key hydrogen-bonds are shown (dotted lines). B) Detailed ligand view of BI8 bound in Eg5. The phenol ring of Tyr352 forms a face-to-face stacking interaction with the benzimidazole ring and makes a water-mediated interaction with a nitrogen atom of the imidazole ring (not shown). Similarly, Tyr104 π-stacks with the trifluoromethylbenzyl motif and a strong hydrogen-bond between the secondary amino group of BI8 and the phenol of Tyr104 can be observed. The inhibitor’s 3-fluoro-4-methoxyphenyl ring is in close proximity to the side chain of Leu292, and Leu295 is buried upon binding. The carboxylate group of BI8 makes a strong hydrogen bond with Thr300, strengthened with a second hydrogen-bond to Arg297 *via* a water molecule (not shown).

Occupying a similar region as the biphenyl compounds, BI8 was found to bind in the pocket formed by α4 of the switch II cluster, α6 which precedes the neck linker region and the β3 strand, with helices α4 and α6 shifting approximately 2Å to accommodate the inhibitor (Figure 11). The neck linker was undocked, but the structure most resembled the intermediate inhibitor-bound state, as previously described for the classical L5/α2/α3 inhibitors. The potency of BI8 for Eg5 could be explained as a result of an extensive network of aromatic interactions.

While there are some similarities between the binding of BI8 and PVZB1194 to Eg5, i.e. shared binding to residues Tyr104, Tyr352, Leu292, there are some striking differences. Notably the neck-linker is docked with PVZB1194 bound and undocked with BI8. The difference in the position of the neck-linker in the inhibitor-bound structure also rendered BI8 different from L5/α2/α3 inhibitors such as STLC, whose crystal structure demonstrated that the final inhibitor-bound state was with the neck-linker in the docked conformation. Additionally, the β3 strand which forms the bottom of the pocket was unaffected by BI8 binding, resulting in an ATP-noncompetitive mode of action [112].

Interestingly, towards the end of the crystal structure refinement for BI8-Eg5, electron density in a site which overlapped both the classical and newly identified allosteric pockets was detected. Parts of the inhibitor in this second site were found to be disordered, which suggested either a greater degree of flexibility and/or lower inhibitor occupancy. However, this raised concerns to whether the majority of potency observed in biochemical assays was due to binding at the α2/α3 site, rather than the crystallographically identified novel a4/a6 site. Deconvolution of the site responsible for the measured inhibition was achieved using two parallel biophysical methods, isothermal titration calorimetry competition and surface plasmon resonance analysis, which confirmed the α4/α6 site to be the single high affinity site in this instance. Measurement of the strength of hydrogen bonding interactions in the α2/α3 site further supported that this was the weaker affinity binding site [112]. Counter-screening of the benzimidazole-based Eg5 inhibitors against HSET highlighted a number with weaker HSET inhibitory activity [113].

## 8 Future Perspectives

The clinical data thus far for Eg5 inhibitors as a monotherapy has been largely disappointing. However, recently published clinical trial data with 4SC-205 showed stable disease was achieved with the Eg5 inhibitor monotherapy dosed continuously, suggesting that refinement of the dosing regimen may be a viable strategy to overcome the low fraction of mitotic cells in slow growing tumours [85]. Tackling this ‘proliferation paradox’ is likely to be important not only for mitotic kinesins, but also for other agents acting predominantly in mitosis.

While some combinations of Eg5 inhibitor with other chemotherapies have also shown limited benefit in clinical trials, e.g. SB-715992 in combination with docetaxel in solid tumours, preclinical studies have suggested that the kinesin inhibitors may be effective in combination with other agents, particularly an Aurora A [91] or KIF15/TPX2 inhibitor [89] should one reach the clinic. Such combinations may provide an opportunity to address functional redundancy among kinesins and improve the efficacy of Eg5 inhibitors. This highlights the requirement for preclinical selection of combination therapies based on mechanistic rationales, and parallels the clinical experience in oncology with targeted agents for signalling kinases. Here, functional redundancy between kinase signalling cascades is a frequent mode of resistance to monotherapy, and may be tackled through drug combinations that simultaneously suppress the alternative pathways [114]. Should Eg5 inhibitors with an alternative modality (e.g. ATP-competitive) reach the clinic, combination of these inhibitors with the classical ATP non-competitive inhibitors (such as ispinesib) may also be of interest. Dual inhibition of Eg5 at both allosteric binding sites may result in increased clinical efficacy by fully arresting the motility of Eg5 along microtubules, and may also reduce the chances of drug-resistant strains of Eg5 emerging by targeting two distinct sites on the same protein simultaneously.

As is the case with many chemotherapeutic strategies against cancer, the development of resistance to Eg5 inhibitors is highly likely, particularly those targeting the L5/α2/α3 binding site. The unpredictable ‘resistance by allostery’ effect observed following inhibition of Eg5 by SB-743921 led to the suggestion that inhibitors occupying an alternative allosteric site may be able to overcome this [95]. Thus, characterization of where and how inhibitors bind and which residues are susceptible to mutation to generate inhibitor resistance is likely to be important in the future development of kinesin inhibitors. The identification of a second allosteric ligand binding site within the Eg5 protein, with chemical tools co-crystallised in the pocket between the α4 and α6 helices, represents a new avenue for the development of inhibitors. Agents targeting this α4/α6 site may not only have improved resilience to the drug resistance mechanisms affecting the L5/α2/α3 allosteric site, but by inhibiting Eg5 in an ATP-competitive manner offer a mode of action which could block, rather than slow, microtubule sliding and lead to a more potent effect in cancer cells [92].

Achieving selectivity for Eg5 over HSET has not proven to be a challenge in inhibitor development at the classical L5/α2/α3 allosteric site, which can be explained by the differences in the L5 region of the two proteins. However, to date no crystallographic evidence of HSET binding is available, therefore the binding sites of the two specific HSET inhibitors AZ82 and CW069 have not yet been elucidated. Interestingly, AZ82 has been identified as an ATP-competitive inhibitor of HSET. As the only ATP-competitive inhibitors of Eg5 identified to date occupy an allosteric site distinct to that of the L5/α2/α3 site, and not from direct interaction with the ATP site, it is possible that binding of the ATP-competitive mode of action of AZ82 also results from structural changes arising from occupation of a novel allosteric site.

The large number of Eg5 clinical candidates spread over several chemical scaffolds and the recent identification of HSET chemical tools, demonstrate the tractability of kinesins to high-throughput screening and structural biology approaches to find and develop inhibitors. This is despite the high degree of flexibility shown by the proteins and the multi-protein nature of their interactions with microtubules. The structural biology elucidation of the complex mechano-chemistry of the Eg5 kinesin has proceeded in tandem with a better understanding of how allosteric inhibitors interfere with the motor and binding functions. The similarities and differences between the druggable sites on Eg5 and HSET are not yet fully understood and inhibitor-bound structural information is likely to be crucial for the development of future HSET and Eg5 clinical candidates, and indeed other mitotic kinesins of pharmacological interest.

## Supplementary Material

Online Appendix

## Figures and Tables

**Table 1:** Structures of Eg5 inhibitors discussed in the text and their biochemical and cellular inhibitory activities

Compound Class	Compound Identifier	Structure	Eg5 Biochemical IC_50_ (nM)^a^	Eg5 Cellular EC_50_ (nM)^b^	References
Dihydropyrimidine-thione	(*S*)-Monastrol **1**	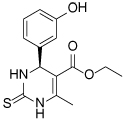	**30000**	**14000**^c^	[42–44]
	*rac*-Mon-97 **2**	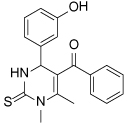	**2(*R*) 150** **2(*S*) 650**	**2(*R*) 1700** **2(*S*) 6500**	[44,45]
	(*S*)-Enastron **3**	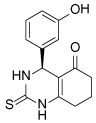	**2000**	**ND**	[42,44]
	(*S*)-Dimethyl enastron **4**	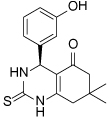	**200**	**ND**	[42,44]
(*R*)-Fluorastrol **5**	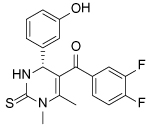	**ND**	**330**	[44]
Tetrahydro-*β*-carboline	**6**	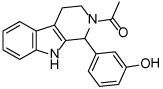	**2500**	**ND**	[46]
	**7**	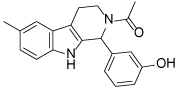	**200**	**ND**	[46]
	**8**	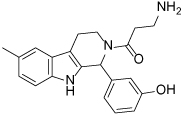	**20**	**83**	[46]
	**9**	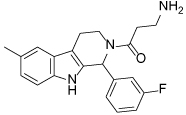	**100**	**ND**	[46]
Tritylcysteine	STLC **10**	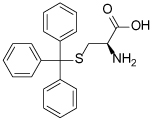	**140**	**700**^d^	[47]
	**11**	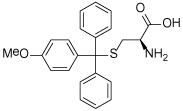	**29**	**ND**	[48]
	**12**	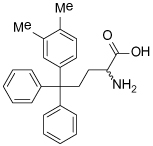	**10**^e^	**28**	[49]
	**13**	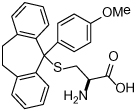	**14**	**28**	[50]
Thiophene-containing	**14**	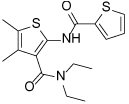	**23000**	>**30000**^f^	[51]
	**15**	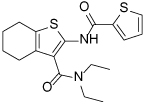	**1700**	**2000**^f^	[51]
	**16**	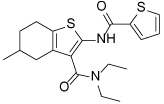	**480**	**1000**^f^	[51]

a Values are IC_50_s in a microtubule-stimulated biochemical assay unless otherwise indicated;

b Effective concentration for 50% growth inhibition in colon cancer HCT116 cells unless otherwise indicated;

c Concentration for 50% inhibition of microtubule motility in mammalian epithelial kidney BS-C-1 cells;

d Concentration for 50% induction of mitotic arrest in HeLa cells;

e MT-stimulated ATPase activity expressed as Ki_app_;

f Effective concentration for 50% growth inhibition due to prolonged mitotic arrest in A-549 hNSCLC cells.

**Table 2:** Published selective inhibitors of HSET and their biological activities

Compound Identifier	Structure	HSET Biochemical IC_50_ (nM)^a^	References
**CW069 17**	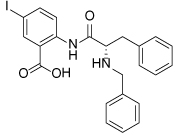	**75000**	[60]
**AZ82 18**	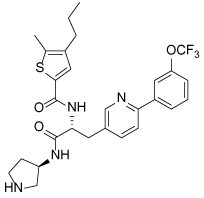	**310**	[61]

a IC_50_s determined in microtubule-stimulated biochemical assays.

**Table 3:** Clinical evaluation of Eg5 inhibitors

Compound Identifiers/Company	Structure	Phase	Clinical trial status^a^
Ispinesib (SB715992) **19** Cytokinetics	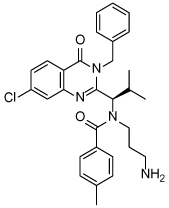	I/II	15 trials completed 1 trial terminated
SB743921 **20** Cytokinetics	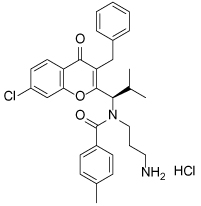	I/II	2 trials completed
AZD4877 **21** AstraZeneca	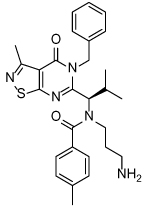	I/II	3 trials completed 3 trials terminated
Arq621 **22** ArQule	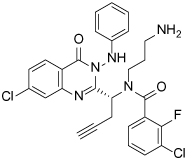	I	1 trial completed
EMD-534085 **23** Merck-KGaA	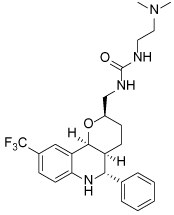	I	Not known b
MK-0731 **24** Merck&co	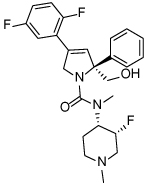	I	1 trial completed
ARRY-520 (Filanesib) **25** Array BioPharma	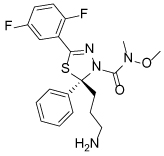	I/II	2 trials completed 4 trials ongoing 2 trials planned
LY2523355 (Litronesib) **26** Kyowa Kakko Kirin/Eli Lily	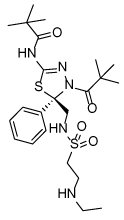	I	6 trials completed 1 trial terminated
4SC-205 4SC AG	Not disclosed	I	1 trial completed

a Data from www.clinicaltrials.gov, accessed 15.10.2015;

b The compound is reported to have entered clinical trials [8,69]

**Table 4:** Eg5 Inhibitors targeting the α4/α6 allosteric site and their biological activities

Compound Identifier	Structure	Eg5 Biochemical IC_50_ (nM)^a^	References
**GSK-1 28**	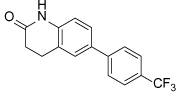	1.8^b^	[106]
**GSK-2 29**	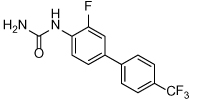	8.8^b^	[106]
**GSK-3 30**	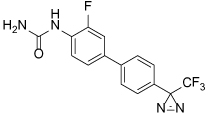	330^b^	[106]
**PVZB1194 31**	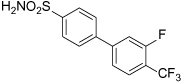	120	[107]
**32**	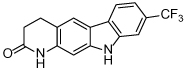	31	[108]
**33**	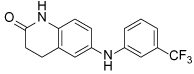	45	[109]
**BI8 34**	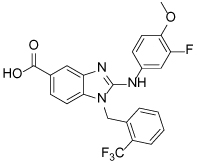	590	[110]
35	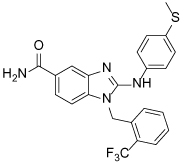	520	[110]

a Biological activity values are IC_50_s determined in microtubule-stimulated biochemical assay unless otherwise stated;

b Microtubule-stimulated ATPase activity expressed as Ki_app_.

## Executive Summary

- Eg5 and HSET interpolar mitotic kinesins have opposing motor functions in mitotic spindle assembly
- Both present attractive cancer cell selective targets
- Structure-based understanding of allosteric inhibition underpins the discovery of kinesin inhibitors
- Current challenges in Eg5 inhibitor clinical development including poor efficacy and resistance may be addressed by changes to drug schedule or inhibitors targeting novel allosteric sites
