# Supplementary material for: Recent findings and future directions for interpolar mitotic kinesin inhibitors in cancer therapy
Source: Future Med Chem. Author manuscript; Available in PMC 2016 Jun 7. (PMC4896392; doi:10.4155/fmc.16.5)
Supplement: Online Appendix [file NIHMS68393-supplement-Online_Appendix.pdf]

Online Appendix

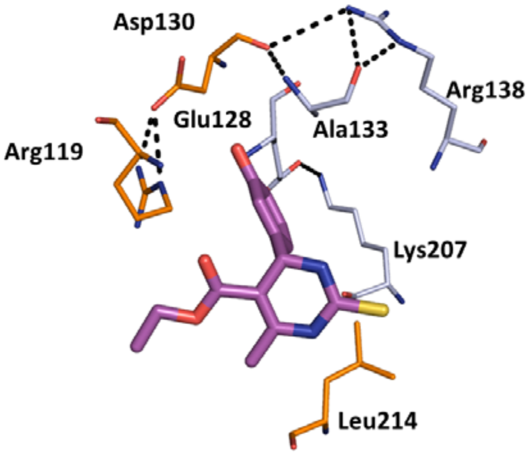

Online Appendix Figure 1: Residues shown in stick form (orange) when mutated confer significant resistance towards the chemical probes monastrol (shown in stick form, magenta) and STLC. Mutation of D130 also confers particular resistance against the clinical candidates ispinesib and SB-743921. Energy gate residue A133 (light blue) is also mutated in a second ispinesib/SB-743921-resistant strain of Eg5. Other relevant residues (shown in light blue) and bridging interactions (dotted lines) affected by mutation of R119, D130 and/or L214 are also shown.
